# Differences in α-Synuclein Conformational States in Physiologically Relevant pH/Na^+^ Concentrations and Ammonium Acetate Solutions Unveiled by Native Mass Spectrometry

**DOI:** 10.1039/d5an01336d

**Published:** 2026-03-17

**Authors:** Erick G. Báez Bolívar, Jessica S. Fortin, Taiwo A. Ademoye, Scott A. McLuckey

**Affiliations:** 1 Department of Chemistry, Purdue University, West Lafayette, IN, USA 47907-2084; 2 Basic Medical Sciences, College of Veterinary Medicine, Purdue University, West Lafayette, IN USA 47907

## Abstract

Native mass spectrometry implemented with theta emitters was used to demonstrate differences in conformational states of wild-type, A53T mutant, and truncated α-synuclein dissolved at physiologically relevant pH and Na^+^ concentrations compared to aqueous solutions of ammonium acetate. Specifically, 150 mM NaCl at pH 7.4, 20 mM NaCl at pH 4.5, and 15 mM NaCl at pH 7.2 were used to reflect, to some extent, the extracellular environment, lysosome, and cytosol, respectively. Analysis of charge state distributions obtained from physiologically relevant solutions vs. their ammonium acetate counterparts allows the comparison of α-synuclein conformational states. The protein shows relatively high conformational flexibility at 150 mM NaCl and pH 7.4, while it shows at least two different conformational states at 20 mM NaCl and pH 4.5. We observed a trend towards the adoption of less compact conformations at acidic pH, where Na^+^ appears to play a distinctive role in the adoption of different conformational states. Early-stage oligomers (dimer, pentamer, hexamer and heptamer) were also detected. Since oligomer formation was protein-specific, wild-type α-synuclein formed dimers while truncated α-synuclein formed pentamers, hexamers and heptamers, their abundances are consistent with kinetics of aggregation reported in the literature.

## Introduction

Many proteins are globally unstructured and are referred to as intrinsically disordered or unstructured proteins (IDP or IUP). The functional importance of IDPs is unquestioned since most of these proteins have basic regulatory roles in key cellular processes. Importantly, IDPs undergo disorder-order transitions according to environmental conditions and binding events. Therefore, they can adopt secondary/tertiary structures that correlate with their specific functions.^1–6^ α-Synuclein (αS) is an IDP that consists of 140 amino acid residues (~14.5 kDa) divided into three domains: the positively charged vesicle-binding N-terminal domain (1–66)^7–9^; the hydrophobic non-amyloid-β component (NAC) domain (67–96), which is essential for nucleation of the aggregation process^10,11^; and the acidic C-terminal domain (97–140), rich in aspartate and glutamate residues.^8,11^ Despite the fact αS is commonly regarded as a monomeric IDP, there is evidence that endogenous, and recombinantly expressed αS is able to form a tetramer under non-denaturing/mild purification conditions.^12,13^

αS is mainly localized in the cytosol of presynaptic nerve terminals, where it controls exocytosis through SNARE-mediated vesicle fusion to the plasma membrane,^14^ and aids in the endocytosis process through clathrin interaction.^14^ Altogether, it regulates vesicle transportation, neurotransmitter release, transmembrane trafficking, and postsynaptic responses.^14,15^ αS aggregates/fibrils are the major component of Lewy bodies, intraneuronal protein deposits commonly found in nerve cells in the brain of patients with Parkinson’s disease (PD), and other sporadic neurogenerative diseases called α-synucleinopathies.^14,15^ PD is a movement disorder of the nervous system. It is the second fastest growing neurological disorder worldwide with 12 million people projected to have this condition by 2040.^16^ Moreover, missense mutations in αS (A30P, E46K, H50Q, G51D, A53E, A53T), and αS gene duplication and triplication, predispose individuals to PD and are, therefore, linked to early onset of the disease.^14,15^ Notably, one of the most common questions is the influence of environmental factors (solution conditions) on the kinetics of fibrillation of αS. The understanding of factors that affect the kinetics of fibrillation might shed light on potential strategies to inhibit PD development (e.g., stabilizing transient folding intermediates).^16,17^ Today, it is known that αS conformational states in solution affect not only the kinetics of fibrillation, but also the fibril morphology. These finding were made via the use of a range of analytical techniques: fluorescence,^18^ nuclear magnetic resonance (NMR),^19^ small-angle x-ray scattering (SAXS),^20^ far-ultraviolet circular dichroism,^21^ Fourier transform infrared spectroscopy,^21^ atomic force microscopy (AFM),^22^ total internal reflection fluorescence microscopy,^23^ Raman spectroscopy;^24^ as well as molecular dynamics simulation.^25^

Native electrospray ionization mass spectrometry (native ESI-MS or native MS) employs conditions that minimally perturb native structures, and inter- and intra-molecular interactions.^26–29^ Information on conformational states of folded and disordered proteins can be obtained by charge state distribution (CSD) analysis.^30–45^ For instance, structural information on αS has been derived using different environmental conditions: presence of ligands, different solution pH, presence of simple and fluorinated alcohols, relatively high solution temperature, and metal ions.^44–53^ The physiological range of pH has been considered (pH 4 – 7.5), but physiological Na^+^ ion concentration mimicking extracellular levels has not been examined. For example, the maximum concentration of Na^+^ ions used to evaluate conformational states of αS was 5 mM.^51^ However, intracellular levels of Na^+^ are 10 – 15 mM and extracellular levels are 135 – 145 mM.^54–57^ Importantly, Na^+^ is the major cation in the extracellular fluid,^54–56^ where αS can be found in a pathological context.^58–60^ The reasons to avoid physiological concentrations of Na^+^ in native MS are simple: salts can give rise to extensive chemical noise, which can interfere with signals originating from ions of interest. Furthermore, metal ions can adduct to the biomolecules of interest resulting in peak broadening, thereby complicating mass determination. In the worst-case scenario, salts can suppress the generation of biological ions of interest by sequestering the available excess charge. Hence, native MS usually relies on one or more desalting steps to remove or reduce non-volatile salts from solution. In general, biological buffers commonly used for protein extraction, purification, and storage are exchanged for ammonium acetate (a MS-compatible volatile salt).^61,62^ Unfortunately, sample desalting can alter protein conformation and dynamics, including equilibria of protein complexes,^63–66^ which complicates the correlation between protein conformational states observed by native MS and its physiological environment. These alterations arise due to the fact that protein conformation does not depend solely on physiological pH or ionic strength but also on the identities of the constituent ions.^67^

The Hofmeister ion series orders cations and anions based on their propensities to either stabilize or destabilize protein structures in solutions.^68^ For instance, the CSD of reduced and alkylated bovine pancreatic ribonuclease A shifted toward lower charge states when 175 mM ammonium acetate (AmAc) was replaced with 150 mM NaCl/25 mM Tris for solutions with the same nominal ionic strength and pH, indicating protein stabilization (folding) in solution.^67^ In contrast, a shift to higher charge states was observed for solutions of bovine serum albumin containing 50 mM sodium acetate compared to 50 mM AmAc, suggesting destabilization (unfolding).^69^ Recently, charge detection mass spectrometry found enhanced adeno-associated virus 9 capsid structural stability in 1X phosphate buffer saline with 350 mM NaCl and 0.01% pluronic F68 compared to 500 mM AmAc in solutions at pH 4 incubated at 37 °C.^70^ These works were possible with the implementation of submicron emitters (internal diameter (i.d.) < 1 μm) to minimize deleterious salt effects on mass spectra by minimizing the size of the ESI droplet.^71^

Here, we use native MS to evaluate conformational states of recombinantly expressed human wild-type (WT) αS and the A53T mutant by following shifts in the CSDs observed in the mass spectra. Considering the cellular context linked to αS, we systematically used a combination of physiological pH, ionic strength, and Na^+^ concentrations to mimic the environment of different cellular locations. We employed a recently developed native MS-based method implemented with theta emitters for the mass analysis of proteins and protein complexes from common biological buffers containing physiologically relevant Na^+^ concentrations.^72,73^ Briefly, the method relies on the manipulation of the composition of the ESI droplets, yielding a fraction of droplets that are relatively depleted in non-volatile salts that gives rise to the resolved analyte charge states. Of particular note, the conformational changes interpreted from the mass spectra correlate with in-solution assays reported in the literature (see below). We acknowledge that the conformational landscape of αS is not only affected by the ionic strength of the solution^74^ and the presence of metal ions^45,48,51,52,74^, it is also modulated by a range of ubiquitous charged biopolymers,^46,75^ where glycosaminoglycans are of particular importance.^76,77^ However, the evaluation of conformational changes of αS dissolved in physiologically relevant Na^+^ concentrations in presence of ligands falls outside the scope of this work.

## Results and Discussion

αS is mainly located in the cytoplasm,^14,15^ but it has been found in the extracellular environment^58–60^ and in lysosomes^78,79^. Therefore, a combination of physiological pH and Na^+^ concentrations were used to reflect those specific environments. For instance, 150 mM NaCl/pH 7.4^80^, 15 mM NaCl/pH 7.2^81^, and 20 mM NaCl^57^/pH 4.5^82^ were selected to mimic the extracellular environment, the cytosol, and the lysosome, respectively. 25 mM Tris buffer was used to regulate solution pH around 7.2 – 7.4, while glacial acetic acid was used to create the acetate buffer at pH 4.5. The spectra of the protein dissolved in physiologically relevant conditions (e.g., 150 mM NaCl/pH 7.4) were compared to their AmAc counterparts (e.g., 150 mM AmAc/pH 7.4). Noteworthy, this work does not intend to replicate spectra reported in the literature since the use of physiologically relevant Na^+^ concentrations coupled to the addition of biological buffers (e.g., Tris buffer) had not previously been considered. Instead, AmAc solutions of 10 mM,^46,48^ 20 mM,^45,51,52^ and 50 mM^49,50^ are common solution conditions. Commercial human WT αS (referred to herein as αS) and αS with an A53T mutation (mαS) were used as model systems as the latter has shown faster fibrillation kinetics (Figure S1).^18,24^ Additionally, αS in-house produced was chosen as a model system because it has shown lower solution stability than the commercial version. This is presumably due to differences in the expression and/or purification process.^83,84^ The company considered this information as proprietary when consulted.

**Figure 1.**
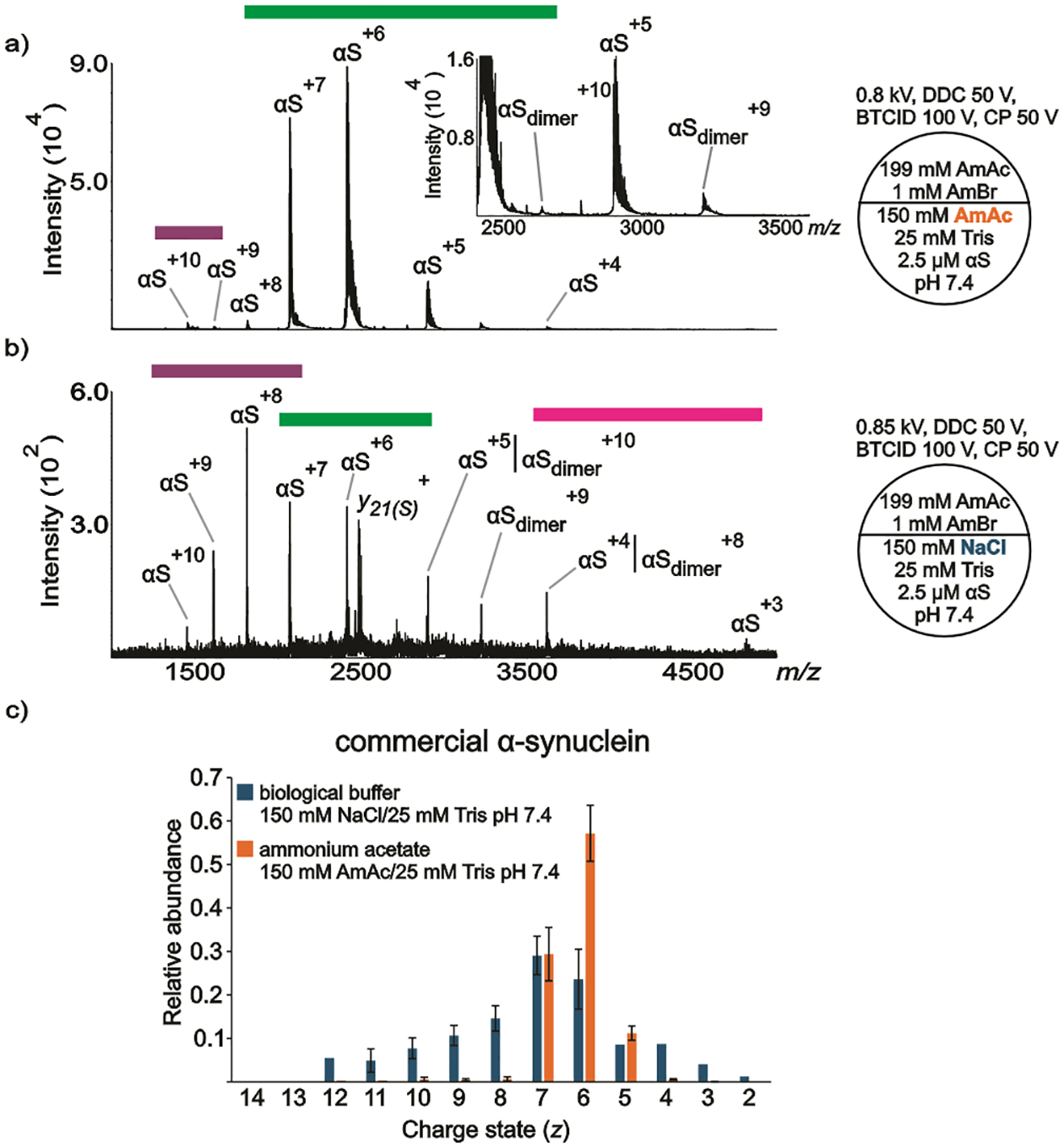
Representative nESI mass spectra acquired in positive ion mode for aqueous solutions of commercially available a) αS (2.5 μM) dissolved in 150 mM AmAc plus 25 mM Tris pH 7.4, b) αS (2.5 μM) dissolved in 150 mM NaCl plus 25 mM Tris pH 7.4, c) relative abundances of the ion peaks observed in “a” and “b”. Three independent replicates were taken into consideration. The circle split by half represents a theta emitter. The purple, green and fuchsia bands, point out the extended, intermediate, and compact populations, respectively.

**Figure 2.**
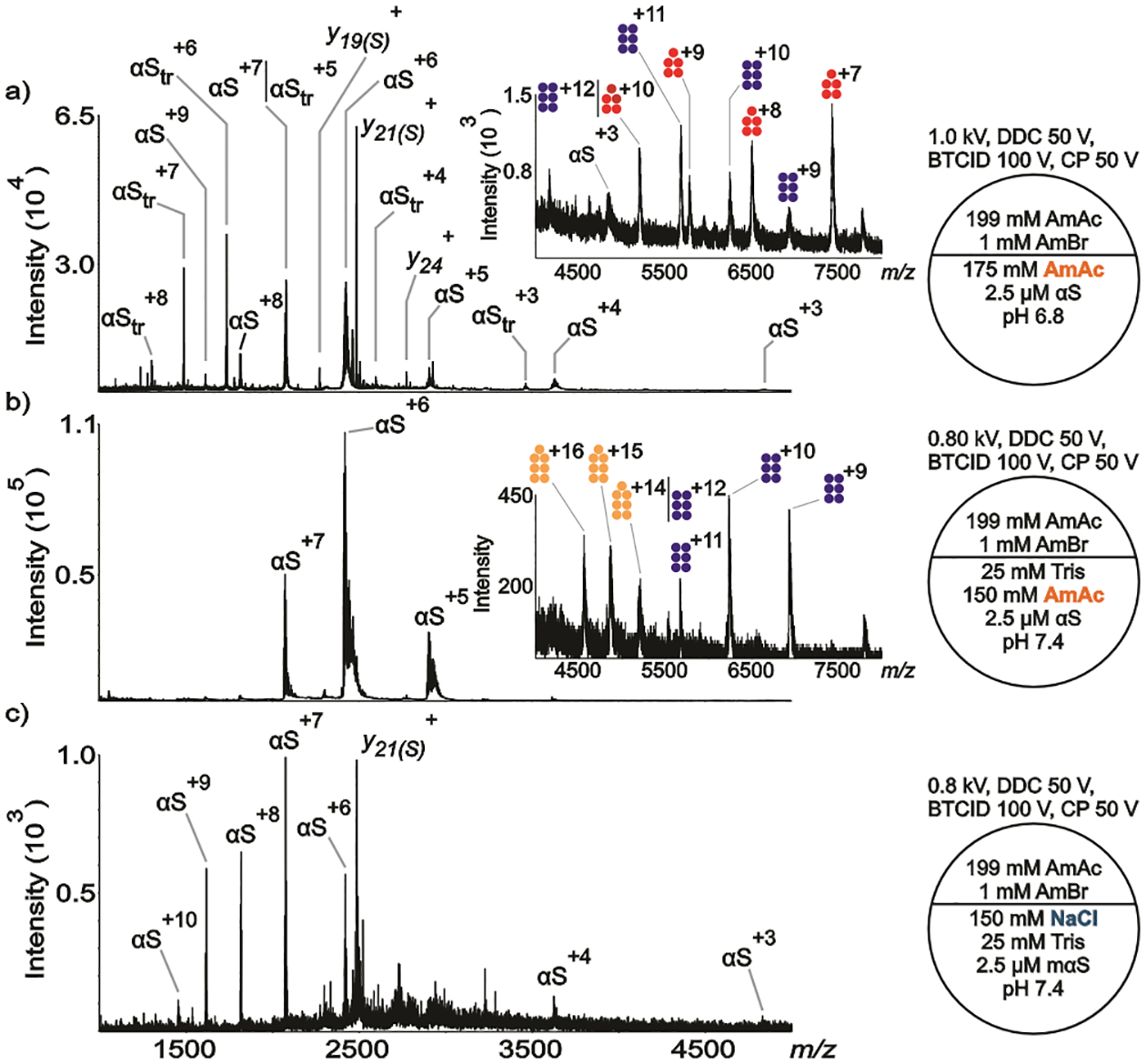
Representative nESI mass spectra acquired in positive ion mode for aqueous solutions of in-house produced a) αS (2.5 μM) dissolved in 175 mM AmAc pH 6.8, b) αS (2.5 μM) dissolved in 150 mM AmAc plus 25 mM Tris pH 7.4, c) αS (2.5 μM) dissolved in 150 mM NaCl plus 25 mM Tris pH 7.4. The circle split by half represents a theta emitter. Pentamer, hexamer, and heptamer of αS_tr_ are represented in fuchsia, blue, and light orange, respectively.

**Figure 3.**
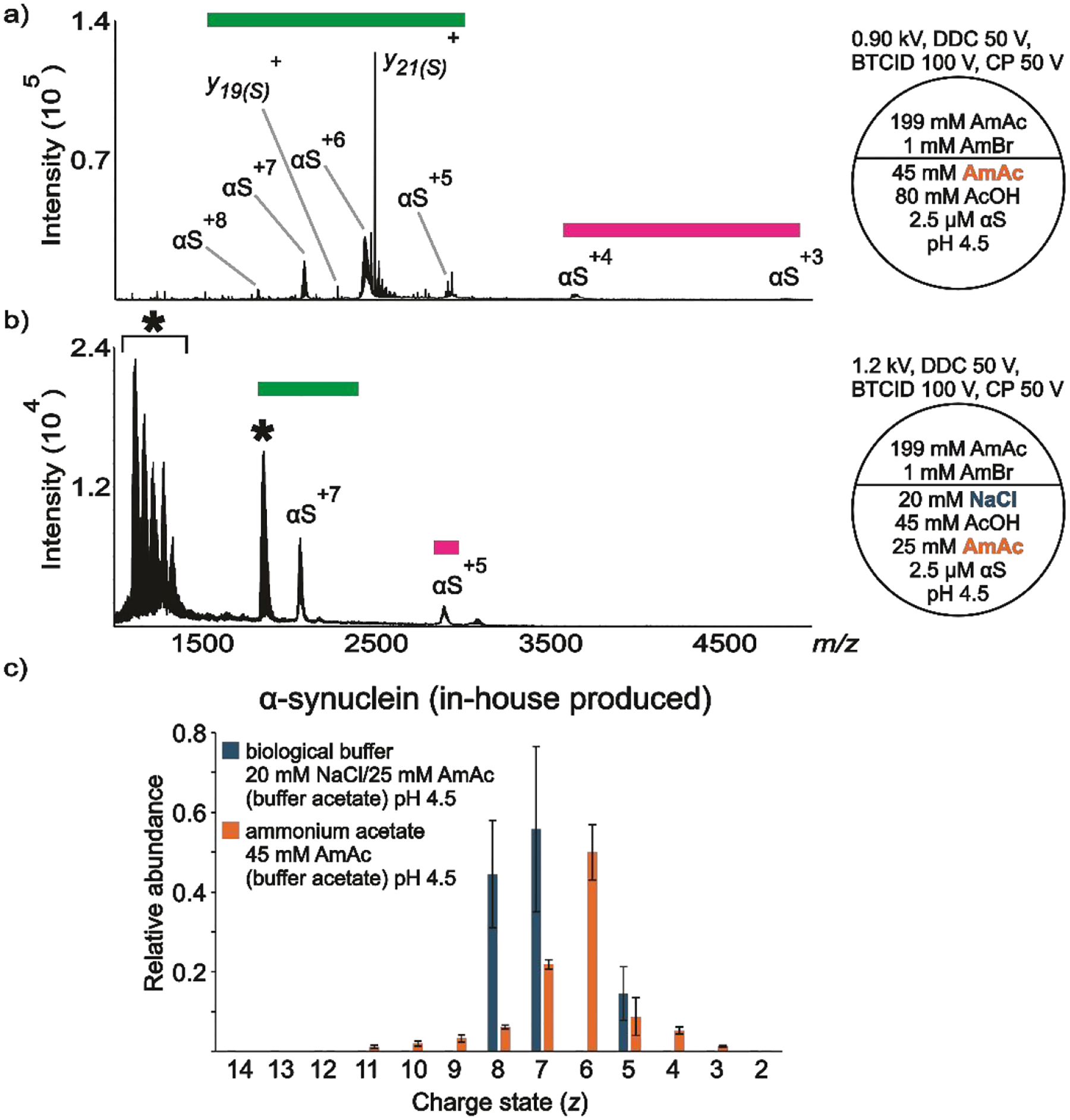
nESI mass spectra acquired in positive ion mode for aqueous solutions of in-house produced a) αS (2.5 μM) dissolved in 45 mM AmAc plus 80 mM AcOH pH 4.5, b) αS (2.5 μM) dissolved in 20 mM NaCl, 25 mM AmAc plus 45 mM AcOH pH 4.5, c) relative abundances of the ion peaks observed in “a” and “b”. Three independent replicates were taken into consideration. The circle split by half represents a theta emitter. The asterisk signals unresolved features in the spectra.

**Figure 4.**
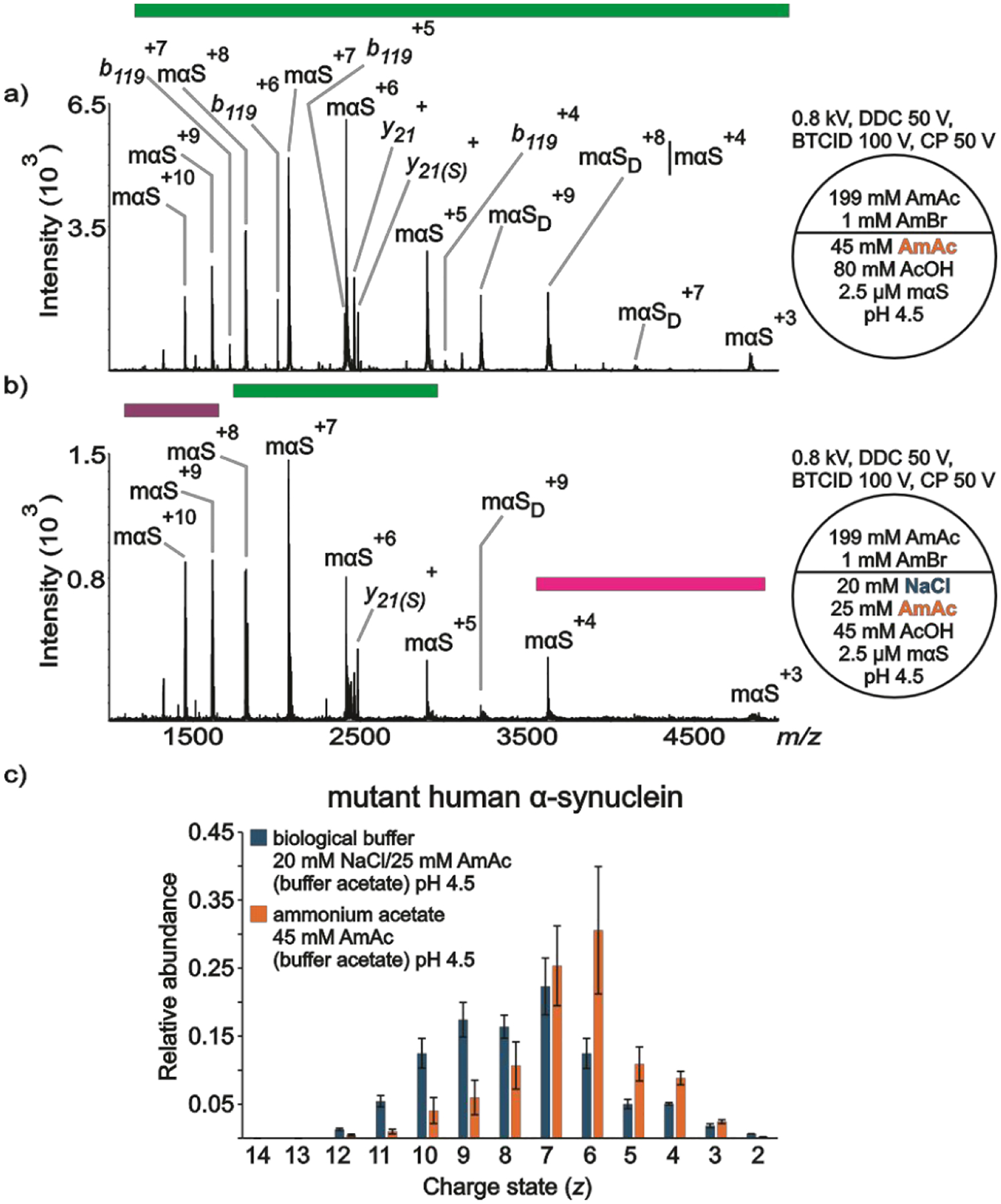
nESI mass spectra acquired in positive ion mode for aqueous solutions of a) mαS (2.5 μM) dissolved in 45 mM AmAc plus 80 mM AcOH pH 4.5, b) mαS (2.5 μM) dissolved in 20 mM NaCl, 25 mM AmAc plus 45 mM AcOH pH 4.5, c) relative abundances of the ion peaks observed in “a” and “b”. Three independent replicates were taken into consideration. The circle split by half represents a theta emitter.

The conformational states of the ion populations are categorized here as extended (purple band), intermediate (green band), and compact (fuchsia band) (see Figure 1). The criterion for assignment of conformational states, specifically between extended and intermediate, is the evidence of local maxima among the charge states observed in the spectra. For example, a typical CSD follows a gaussian curve with a local maximum (e.g., a dominant charge state). The appearance of another ion population in the same spectrum can lead to another local maximum. Unfortunately, the local maxima are not always visible within a convoluted CSD, their visibilities depend on relative ion abundances.^85^ Moreover, αS monomers are conformationally heterogeneous, the identification of poorly populated conformers might have escaped detection.^74^ The assignment of a compact conformation follows the observation of charge state (*z*) = 3 or a local maximum at *z* = 4. The bar plots were created by Excel using the ion intensities of the centroids at each charge state in the spectra from independent replicates. To account for metal ion adduction, the spectrum was smoothed (which covers isotopically unresolved, adducted and/or solvated protein charge states). In the gas-phase conditions noted (Figure 1–4), the first potential (in kV) refers to the potential of the theta tip nESI emitter, DDC is dipolar direct current, BTCID is beam-type collision induced dissociation, and CP is the potential of the curtain plate in front of the nESI emitter (see Mass Spectrometry section in Supplementary Information). The gas phase methods (BTCID, DDC) and the gas phase parameter (CP) aid in the dissociation of metal ion adduction and desolvation.^72,73^ More details about data collection and processing, protein preparation, and chemicals can be found in Supplementary Information.

We note that an ESI droplet lifetime of 27 μs to 270 ns was calculated using theta nESI emitters of i.d. ~1.6 μm (based on the measured outer diameter and outer wall thickness of the emitters),^86^ which is considerably shorter than the time required (500 μs) by αS to fold into a transitional conformation in presence of 1.2 mM sodium dodecyl sulfate (a lipid mimic).^87^ The latter measurement was performed using single-molecule fluorescence.^87,88^ Furthermore, within the conformational ensemble, some conformers have exhibited conformational transitions slower than milliseconds.^89^ Therefore, it is unlikely that the CSDs are determined by structural changes that take place in the ESI droplet. This conclusion is consistent with recent findings for cytochrome *c*, a small globular protein with marked differences in mass spectra between native and denatured states, that indicated kinetically trapped conformation states in ESI droplets.^38^

### 150 mM NaCl at pH 7.4 (extracellular environment)

#### Commercially-produced αS.

Figure 1a shows a representative nanoESI (nESI) mass spectrum of 2.5 μM αS (commercial) dissolved in 150 mM AmAc at pH 7.4. There are two CSDs observed in the spectrum, indicating extended and intermediate ion populations. The extended ion population is represented by αS^(+z) = 9−10^, where αS^+10^ represents the local maximum. This is a very low abundance population. The most abundant ion population is represented by an intermediate conformation with a local maximum at αS^+6^. The intermediate population is dominated by three charge states, αS^(+z) = 5–7^. Despite the low concentration of protein used in these experiments, a relatively low abundant dimeric population (αS_dimer_) is also observed in the spectrum. Figure 1b shows a representative nESI mass spectrum of a solution of 2.5 μM αS (commercial) dissolved in 150 mM NaCl at pH 7.4. In contrast to the spectrum shown in Figure 1a, there are three distinct ion populations observed in the spectrum. Notably, the extended ion population has a higher relative abundance compared to the intermediate ion population. The local maxima are centered at αS^+8^ and αS^+6^ for the extended and intermediate populations, respectively. Despite a noticeable level of chemical noise, αS^+3^ can be assigned in the spectrum. Another contrasting difference relies on the absolute signal intensities, the ion intensities in the spectrum obtained from the solution containing the non-volatile salt (NaCl) can be as much as 150 times lower than the signal ion intensities of the solution without NaCl, which is due to ionization suppression and/or chemical noise.^72,73^ There is also a peak in many of the spectra shown here (*m/z* 2484.98), which is assigned as the sodiated *y*_*21*_ ion (*y*_*21*_(*S*)) (*y*_*21*_+*Na*)^+^, *m/z*_theoretical_ 2484.93. While the presence of a contaminant species cannot be precluded as the origin of this peak, previous MS/MS results for αS show a highly prominent cleavage between the adjacent aspartic acid and proline sites (see Scheme S1) that give rise to a *y*_*21*_/b_119_ complementary pair.^90,91^ Both the sodiated (*m/z* 2484.92, −3 ppm) and non-sodiated (*m/z* 2462.93, *m/z*_theoretical_ 2462.95, −5 ppm) *y*_*21*_^+^ fragment ions are observed in solutions without NaCl with relative abundances of roughly 2% and 3%, respectively. In some of the spectra provided in Supplemental Information, there are also small signals corresponding to sodiated *y*_*24*_ (N-terminal to a proline residue) and sodiated *y*_*19*_ (C-terminal to an aspartic acid residue). Collectively, these results suggest that some gas phase cleavage of the ions occurs under the ion transmission conditions used here. The extent to which this occurs depends upon solution conditions as variable extents of adduction are expected with the different solution conditions used in this work. Conditions that lead to more extensive clustering with ammonium bromide (AmBr), for example, tend to protect the protein from cleavage as energetic collisions that occur in the process of ion transmission tend to drive off salt adducts, rather than cleave covalent bonds. Overall, a significantly more heterogeneous ion population was observed from solutions of protein containing 150 mM NaCl (Figure 1c, Figure S2). αS_dimer_ can be also observed in the spectrum, which contributes to a certain degree of overestimation for αS^(+z) = 4–5^.

Similarly, the relative abundances of the peaks in the spectra collected with mαS and αS in-house produced dissolved in 150 mM NaCl at pH 7.4 reflect a more heterogeneous population in comparison with the same protein dissolved in 150 mM AmAc at pH 7.4 (Figure S3–S6). Notably, the CSD observed in Figure 1a is representative of an ionization process that follows the charge residue mechanism (CRM),^92,93^ where charges are transferred from the evaporating water layer onto the protein surface. Since multiple stages of droplet evaporation and droplet fission are carried out (ejecting protons and small ions during the entire process), relatively fewer charges are available for protein ionization. Hence, the protein ion shows a relatively narrow distribution. In contrast, Figure 1b shows evidence of the chain ejection model (CEM).^94^ In this model, the unfolded polypeptide backbone migrates towards the surface of the droplet due to exposure of hydrophobic residues and presence of an intense electric field. The positioning of the protein at the air-droplet interface, the presence of the electric field, and the charge repulsion at the droplet surface promote an ejection of the protein with simultaneous charge partitioning. Since this is a relatively young ESI droplet, the protein acquires a relatively high number of charges. Thus, the protein ion shows a much wider CSD. Although, we cannot rule out multiple ionization mechanisms occurring simultaneously (CRM and CEM), which has been previously suggested,^50^ the mechanisms still rely on differences in protein conformation in solution. Here, a solution condition mimicking a physiological level of Na^+^ (e.g., 150 mM in the extracellular environment) unambiguously shows αS adopts different conformation compared to a solution with the same nominal ionic strength but with ammonium acetate instead of NaCl.

It is known that the conformation of monomeric αS and the structure of its amyloids depends strongly on the ionic strength of the buffer solution.^95^ Despite the fact that the ionic strengths of the experiments summarized in Figure 1 are the same (175 mM), it is evident that the relative abundances of the ions observed in the spectra containing NaCl are not the same in comparison to the spectra obtained from the solution without NaCl. Importantly, the evidence of solution condition-dependent conformational states observed for αS in the gas phase has been demonstrated in bulk solution as well. In-solution assays, such as different modalities of NMR, have shown that transverse relaxation rate (*R*_2_) values in the absence of NaCl are larger than those in the presence of 150 mM NaCl for both termini, suggesting that the N- and C-termini of αS undergo restricted motion in the absence of NaCl.^19^ To probe salt effects on αS tertiary structure, the authors covalently labeled αS with radical nitroxide (a paramagnetic radical) to extract information about long-range interactions (up to 25 Å) with nuclei. The distance from the spin labeled site was shorter in absence of NaCl (different label sites were tested) compared to 150 mM NaCl, suggesting that the protein adopts more compact conformations in the absence of NaCl.^19^ Moreover, the hydrodynamic radius (*R*_h_) value in the absence of NaCl (*R*_h_ = 27.9 Å) is smaller than that in the presence of 150 mM NaCl (*R*_h_ = 30.4 Å).

SAXS can be used to characterize protein structures in solutions via the measurement of the radius of gyration (*R*_g_). *R*_g_ is sensitive to shapes and binding stoichiometry of proteins. The *R*_g_ values of αS dissolved in 150 mM NaCl at pH 7.4 and a solution of pH 7.4 without NaCl were 42 Å and 27 Å, respectively. This indicates that αS adopts more compact structures at pH 7.4 in solution devoid of NaCl, while it shows the least folded structures at 150 mM NaCl pH 7.4.^20^ To rationalize conformational changes of αS in different NaCl concentrations, molecular simulation dynamics have been implemented.^25^ The authors concluded that the C-terminal domain preferentially interacts with Na^+^, due to its high negative charge, and becomes effectively neutralized at moderate NaCl concentrations (e.g., 150 mM NaCl). This weakens the attractive electrostatic interactions between the C-terminal with the N-terminal and NAC domains (long-range interactions), known to be essential to regulate αS aggregation, and induces extended, solvent-exposed conformations with fewer intra-molecular contacts, which readily form fibrils.^25^

The conclusions from molecular simulation dynamics are consistent with Thioflavin T (ThT) fluorescence results. ThT fluorescence detection is widely used for the detection of amyloid fibrils because ThT binds with relatively high affinity to amyloid structures (*K*_d_ in the low μM),^96^ and the fluorescence intensity is enhanced in presence of fibrils.^96–98^ ThT fluorescence detection has been used to demonstrate that the kinetics of fibrillization of αS are faster in presence of 150 mM NaCl at pH 7.4 compared to a solution of the same pH but without salt, indicating a more aggregation-competent conformation in presence of NaCl.^20,24^ Collectively, the NMR, SAXS, and ThT fluorescence results are consistent with the observations related in Figure 1.

#### In-house produced αS.

The commercially obtained αS and the in-house produced αS showed both similarities and differences in behavior when subjected to very similar conditions. The major difference was that two species were found in the solution with αS in-house produced dissolved in 175 mM AmAc at pH 6.8 (Figure 2a); viz., a full-length αS (14462 ± 1 Da) and a truncated version (αS_tr_, 10408.9 ± 0.1 Da). Little evidence for αS_tr_ was observed with the commercial version (compare Figure S2a). Truncated versions of αS have been previously reported in the literature. For example, αS_(40–140)_ (10437 Da) was detected by MS after protein expression and purification.^99^ All modifications were assigned as truncation at the N-terminus of the full-length protein.^99^ αS_tr_ forms a pentamer (51980 ± 57 Da) and a hexamer (62466 ± 82 Da) in 175 mM AmAc at pH 6.8 (see inset in Figure 2a). The solution comprised of the in-house αS in 150 mM AmAc/25 mM Tris at pH 7.4 showed very little evidence for αS_tr_ monomer (Figure 2b), which is similar to Figure 1a, but small signals consistent with αS_tr_ hexamer (62369 ± 5 Da) and heptamer (73030 ± 170 Da, using UniDec^100^) were observed (see inset in Figure 2b). The observation of strong αS_tr_ monomer signals in Figure 2a but not in Figure 2b could either be due to difference in pH or the presence/absence of buffer. As described below, in the discussion of Figure 3, low pH alone appears not to lead to the observation of αS_tr_. Biological buffers are known to have specific effects in biological systems.^101^ Tris buffer at pH 7.0 has been shown to accelerate the nucleation process of *Escherichia coli* protein RecA on double-stranded DNA.^102^ Moreover, interferon-tau displayed different aggregation rates depending on the nature of the buffer, according to the decreasing series: phosphate > Tris > histidine.^103^ The addition of Tris might explain the presence of higher-order oligomers in Figure 2b compared to Figure 2a. Also, the data suggest that the presence of the buffer influences the conformational landscape of WT and mαS since the protein shows a more heterogeneous population in absence of the buffer when no NaCl is present in solution (Figure 1a, 2b vs Figure S2a; Figure S3e vs S3f–h, Figure S5e vs S5f–h). Additionally, the undetected presence of full-length αS oligomers in the spectra can be rationalized based on differences in aggregation kinetics. Truncations at the N-terminus have shown faster aggregation kinetics in comparison to the full-length protein. Specifically, the time required to reach half of the maximum ThT intensity, *t*_1/2_ (h), values for 70 μM of αS, αS_14–140_, αS_36–140_, and αS_41–140_ are 33 ± 4, 25 ± 3, 20 ± 2, 19 ± 2.^104^ However, C-terminal truncations and post-translational modifications (e.g. phosphorylation) are also associated with PD due to their accumulation in Lewis bodies (suggesting faster aggregation kinetics).^104,105^ Although N-terminal truncations have been used here as a plausible explanation, C-terminal truncations cannot be ruled out. Since we are only detecting oligomers from the truncated protein, our data suggest that αS_tr_ has an apparently higher rate of aggregation compared to αS, and the aggregation of αS_tr_ is not driven by the ionic strength of the solution (compare solution conditions of Figure 2a vs Figure 2b). Clear evidence for αS_tr_ monomer is not apparent in solutions containing 150 mM NaCl/25 mM Tris at pH 7.4 (Figure 2c) and neither are signals from oligomers, although small signals from the latter may be obscured by the chemical noise observed in the spectrum. The general tendency noted in Figure 1 for higher charge states arising in the solution with NaCl (Figure 1b) than with AmAc (Figure 1a) is also observed in the comparison of Figure 2b with Figure 2c.

#### Mutant αS.

The results for the A53T mutant under the solution conditions consistent with the extracellular environment are provided in Figure S5. It shows the same conformational behavior that commercially-produced αS, and in-house produced αS. The larger *R*_h_ value for the mutant protein in the presence of 150 mM NaCl, noted above for the wild-type protein, was also observed for the A53T mutant.^19^ The *R*_h_ represents the average sampling of the protein conformational states in solution, hence the results are consistent with Figure 1c, Figure S4, and Figure S6 for commercial αS, in-house produced αS, and A53T mutant, respectively, where the protein appears to be conformationally flexible in presence of 150 mM NaCl while one main conformation (compact) is observed without NaCl. Notably, there are variations in the relative intensities of the charge states between the individual replicates shown in Figure S2, S3, S5. This has been previously observed for αS dissolved in AmAc solutions, albeit at much lower concentrations,^49,50,106^ and has been rationalized as a shallow conformer landscape where there are few energetic constraints preventing access to multiple conformers.^49,50^ Interestingly, the protein dissolved in relatively high concentration of AmAc (e.g. 150 mM) in absence or presence of the buffer shows more Na^+^ adduction compared to solution conditions where relatively low concentration of AmAc was employed (e.g., 15 mM, 45 mM). Since the protein stock solution was diafiltrated with ultrapure water, we presume the main source of contamination to be the bottle of AmAc.

### In 20 mM NaCl at pH 4.5 (lysosome)

#### Commercial and in-house αS.

The commercially available and in-house produced αS showed very similar behavior under the simulated lysosome conditions (see Figures S7 and S8 for the commercially obtained αS). Figure 3a shows a representative nESI mass spectrum of αS in-house produced dissolved in 45 mM AmAc at pH 4.5. No evidence for αS_tr_ monomer or oligomers is present in either of the spectra obtained at this pH and ionic strength. Two conformational states are present in the spectrum, although αS^+3^ has relatively low abundance. Remarkably, the molecule appears to adopt partially folded conformations at pH 4.5/20 mM NaCl compared to pH 7.4/150 mM NaCl (Figure 3b vs Figure 1b, 2c), which has been previously reported using ion mobility separation and CSD analysis (no Na^+^ added).^107,108^ This observation agrees with FTIR results suggesting differences in β-structure at pH 7.5 vs pH 3.0. It was found that “natively unfolded αS” is transformed into a partially folded conformation with a significant amount of β-structure at acidic pH (no Na^+^ added).^21^ The data were also supported with the measurement of *R*_g_ at neutral (40 ± 1 Å) and acidic pH (30 ± 1 Å).^21^ Conversely, the appearance of two distinct ion populations when the protein is dissolved in NaCl is evident from the nESI spectrum of the protein dissolved in 20 mM NaCl/25 mM AmAc at pH 4.5 (Figure 3b). The assumption of two distinct ion populations is based on the complete absence of αS^+6^ in the spectrum, which consequently generates two local maxima. Also, there are unresolved features in the spectrum. We speculate the formation of clusters and aggregates may account for these signals.^109^ Unfortunately, the study of αS oligomerization falls outside the scope of this work. Therefore, no experiments were designed to understand the nature of the consistent unresolved feature around *m/z* 1850 (Figure S8, S9). Figure 3c shows the relative abundance of the peaks of the protein dissolved in the two solution conditions.

#### Mutant αS.

Figure 4 summarizes data acquired for mαS under conditions intended to simulate the lysosome environment. Figure 4a shows a wide CSD of mαS ions from a solution of the protein dissolved in 45 mM AmAc at pH 4.5. One conformational state was identified in the spectrum (intermediate). Dimeric mαS (mαS_dimer_) was also identified. There are other peaks that appear to be complementary fragments of mαS (b119/y21) that presumably originate from gas-phase fragmentation during ion transmission (b_119_ (12025 Da) + y_21_ (2463 Da) = 14488 Da). The complementary pair does not consistently appear in the spectra (see Figure S10, for example), which may be due to different extents of adduction from one experiment to the next. Figure 4b shows three different conformational states. The local maxima of the extended, intermediate and compact conformational states are centered at αS^(+z) = 9−10^, αS^+7^, and αS^+4^, respectively. However, there could be some degree of contribution from mαS_dimer_^+8^ to mαS^+4^, which appears at the same *m/z*. Figure 4c shows at least two clear CSDs from the plot of relative abundances, one centered at *z* = 9 and the other at *z* = 7 from the solution of the protein containing NaCl. This is in line with the results obtained with the commercially available and the in-house produced versions. However, in contrast with Figure 3, no charge states are absent in the spectra obtained with the mutant version. We also noticed less metal ion adduction onto the A53T mutant compared to other versions tested in this work under similar conditions (Figure S10 vs Figure S8, S9). Although the experiments for each version of the protein were executed the same day, each version could have been tested in different days. Therefore, differences could reflect inconsistencies in instrument performance and/or intrinsic theta nanoESI emitter variability, which has been previously discussed.^73^

Under 20 mM NaCl and pH 5 conditions, αS showed slower kinetics of fibrillation compared to A53T mutant (τ_1/2_ = 3.7 ± 0.7 h vs 1.5 ± 0.3 h, respectively).^24^ Based on spectroscopic features, differences in the overall β-sheet content of the fibrils and in the side chain packing within the fibril structure were elucidated.^24^ Indeed, using a model based on data obtained by microelectron diffraction, the atomic structure of an 11-residue segment of αS suggested that the A53T mutation would influence side-chain packing and lead to a more stable β-sheet interface.^110^ Although we cannot pinpoint specific secondary structures, our data suggest that the conformational dynamics of mαS (A53T mutation) are intrinsically different from those of αS at acidic pH (Figure 3c vs Figure 4c). For instance, there are two contiguous or overlapping CSDs for mαS (Figure 4c) while the CSDs are defined by the absence of αS^+6^ in the WT protein (Figure 3c). Thus, we reason that the differences in CSDs truly mirror differences of conformational states between αS and mαS in acidic solution.

### 15 mM NaCl at pH 7.2 (cytosol)

#### Commercial αS, in-house αS, and mαS.

**Figure 5.**
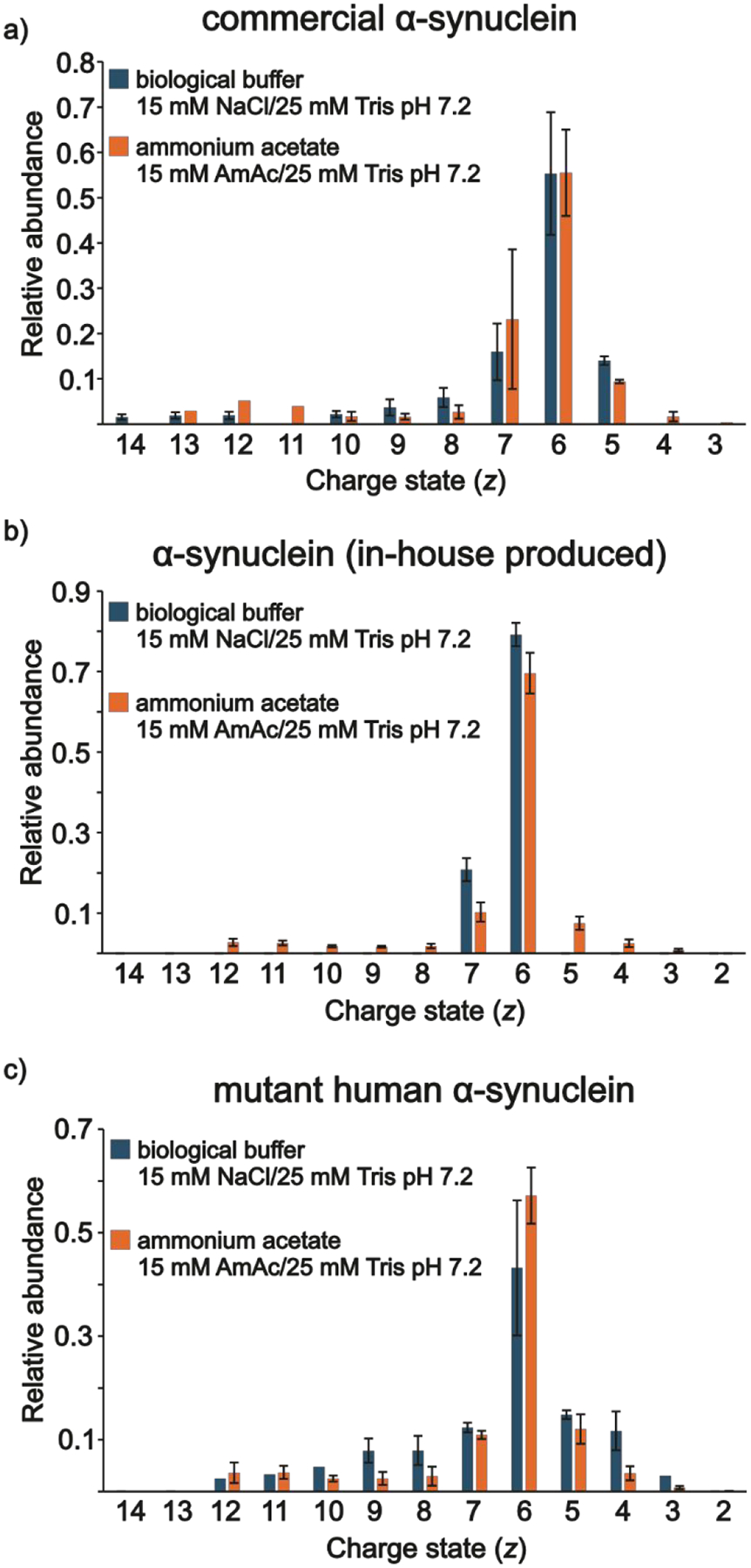
Relative abundances of a) commercial αS_monomer_, b) in-house αS_monomer_, and c) mαS_monomer_ under conditions of 15 mM AmAc and 25 mM Tris at pH 7.2 (orange bars) and under conditions of 15 mM NaCl and 25 mM Tris at pH 7.2 (blue bars).

**Figure 6.**
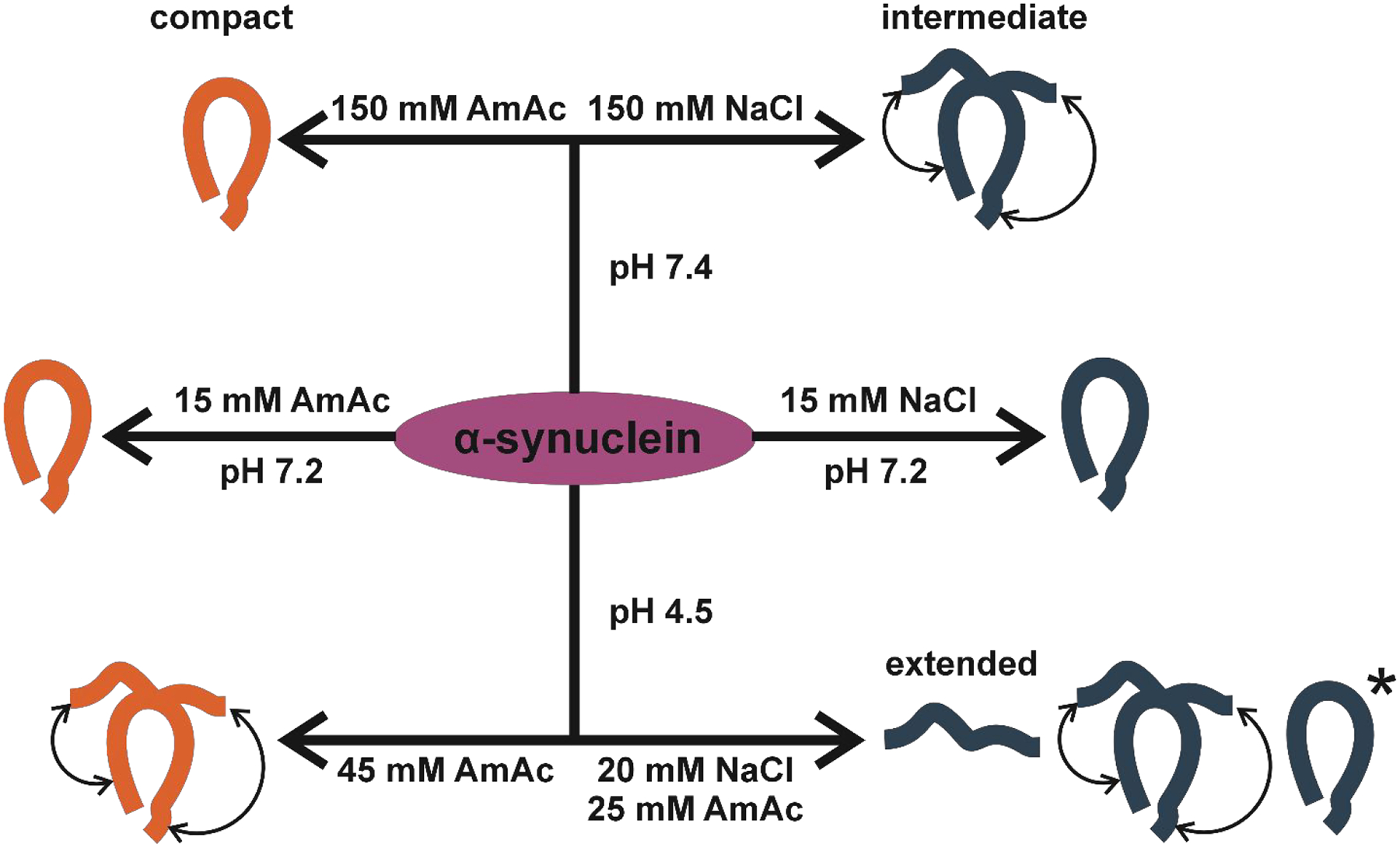
Illustration of the conformers identified in this work as per solution condition. The asterisk represents the distinctive compact conformations observed under acidic conditions.

No clear evidence for differences in conformational states, based on CSDs, were observed from solutions of the proteins dissolved in 15 mM NaCl at pH 7.2 in comparison to their AmAc counterparts for the commercial (Figure 5a) and in-house (Figure 5b) versions of αS. Figure 5 shows the relative abundances of the peaks for the three analytes, which are based on the spectra in Figures S11–S13. The mutant form showed a subtle increase in the relative abundances of higher charge states but not as extensive as with the conditions for the extracellular matrix (Figure S5) or lysosome (Figure 4c). This seems to support the previous observations in the extracellular and lysosome solution conditions. In the former, ample conformational flexibility were noted due to the relatively high concentration of Na^+^, which interaction with the negatively charged C-terminus occludes intramolecular long-range interactions, allowing the relaxation of the molecule.^19,25^ In the latter, the relatively low pH promotes the adoption of two distinct partially folded conformations, which has been previously reported using 10 mM AmAc (no added Na^+^).^108^ However, NaCl does seem to play a role. Again, here, a moderate pH and Na^+^ concentration leads to a negligible change in protein conformation compared to similar solution conditions but in the absence of NaCl. This is in agreement with molecular dynamics solution indicating that at low NaCl concentrations long-range interactions are not effectively neutralized.^25^ Figure 6 shows an illustration of our findings.

## Conclusions

Using native mass spectrometry implemented with theta emitters, we demonstrated differences in conformational states of α-synuclein dissolved in physiological pH and Na^+^ concentrations compared to solutions containing ammonium acetate. In this work, we simulated physiological Na^+^ concentrations and pH values found in three different cellular contexts that α-synuclein resides, including the extracellular environment (150 mM NaCl pH 7.4), lysosome (20 mM NaCl pH 4.5), and cytosol (15 mM NaCl at pH 7.2). Solutions reflecting the extracellular environment and lysosome promoted different α-synuclein conformational states compared to their ammonium acetate counterparts. Notably, the conformational flexibility in 150 mM NaCl pH 7.4, and the adoption of distinct conformational states in 20 mM NaCl pH 4.5 reflected in the CSDs observed in the mass spectra, correlate with in-solution assays performed under similar physiological conditions. Furthermore, early-stage oligomers (e.g., dimer, pentamer, hexamer, and even heptamer) were detected in solutions containing truncated α-synuclein and mutant α-synuclein, which are known to have faster kinetics of aggregation in comparison to wild-type. On the other hand, little or no difference in CSDs were noted for spectra obtained using solution conditions simulating the cytosol versus those derived from solutions containing equivalent ammonium acetate concentrations (no added Na^+^).

The significance of this work is that it provides direct mass spectrometric evidence of differences in the conformational states populated by wild-type and mutant αS in solution conditions with physiologically relevant Na^+^ concentrations. While the use of CSDs observed using nESI to draw conclusions regarding conformational states in solution is well-established, the replacement of physiological salts with volatile salts, such as ammonium acetate, to improve mass spectral quality may not lead to CSDs that reflect conformational states under physiological conditions. The theta tip approach used here, which rapidly alters the ESI droplet composition on a timescale shorter than proteins reorganize, enables interpretable mass spectra to be obtained from solutions at physiological pH and Na^+^ concentration levels. This approach therefore adds value to the use of native mass spectrometry for the study of the conformational states of intrinsically disordered proteins.

The data supporting this article have been deposited as part of the Supplementary Information.

## Supplementary Material

SI - accepted

## Data Availability

The data supporting this article have been deposited as part of the Supplementary Information.
