# Supplementary material for: Differences in α-Synuclein Conformational States in Physiologically Relevant pH/Na+ Concentrations and Ammonium Acetate Solutions Unveiled by Native Mass Spectrometry
Source: Analyst. Author manuscript; Available in PMC 2026 Mar 20. (PMC12911477; doi:10.1039/d5an01336d)
Supplement: SI - accepted [file NIHMS2149065-supplement-SI_-_accepted.docx]

**Supporting information for:**

**Differences in α-Synuclein Conformational States in Physiologically Relevant pH/Na^+^ Concentrations and Ammonium Acetate Solutions Unveiled by Native Mass Spectrometry**

Erick G. Báez Bolívar^1^, Jessica S. Fortin^2^, Taiwo Ademoyo^2^, Scott A. McLuckey^1^*

*^1^Department of Chemistry, Purdue University, West Lafayette, Indiana, USA 47907-2084*

*^2^Basic Medical Sciences, College of Veterinary Medicine, Purdue University, 625 Harrison Street, West Lafayette IN 47907, USA*

* Corresponding Author:

Dr. Scott A. McLuckey

560 Oval Drive

Department of Chemistry

Purdue University

West Lafayette, IN 47907-2084, USA

Phone: (765) 494-5270

Fax: (765) 494-0239

E-mail: [mcluckey@purdue.edu](about:blank)

<https://orcid.org/0000-0002-1648-5570>

Table of Contents

[Experimental section 3](#_Toc215996192)

[Safety comment 3](#_Toc215996193)

[Chemicals 3](#_Toc215996194)

[Preparation of recombinant human α-synuclein (in-house produced αS) 3](#_Toc215996195)

[Proteins 4](#_Toc215996196)

[Mass Spectrometry 4](#_Toc215996197)

[Thioflavin T (ThT) fluorescence assay 6](#_Toc215996198)

[Equation 6](#_Toc215996199)

[Scheme 7](#_Toc215996200)

[Figures 8](#_Toc215996201)

[Figure S1 8](#_Toc215996202)

[Figure S2 9](#_Toc215996203)

[Figure S3 10](#_Toc215996204)

[Figure S4 10](#_Toc215996205)

[Figure S5 11](#_Toc215996206)

[Figure S6 11](#_Toc215996207)

[Figure S7 12](#_Toc215996208)

[Figure S8 13](#_Toc215996209)

[Figure S9 14](#_Toc215996210)

[Figure S10 15](#_Toc215996211)

[Figure S11 16](#_Toc215996212)

[Figure S12 17](#_Toc215996213)

[Figure S13 18](#_Toc215996214)

[References 19](#_Toc215996215)

# Experimental section

## Safety comment

No unexpected or unusually high safety hazards were encountered.

## Chemicals

Trizma Base (Tris, molecular weight (MW) 121.1 Da, purity ≥ 99.9%), ammonium acetate (MW 77.08 Da, purity ≥ 99.99%) were purchased from Sigma-Aldrich (St. Louis, MO, USA). Sodium chloride (MW 58.44 Da, purity 100%) was purchased from Mallinckrodt (Paris, KY, USA). Glacial acetic acid (MW 60.05 Da) was purchased from Mallinckrodt Chemicals (Phillipsburg, NJ, USA). Ammonium bromide (MW 97.95 Da, purity > 99%) was purchased from Sigma-Aldrich (Milwaukee, WI, USA). Water optima LC/MS was purchased from Fisher Chemical (Fair Lawn, NJ, USA). BCA Reagent A, BCA Reagent B were purchased from G-Biosciences (St. Louis, MO, USA).

## Preparation of recombinant human α-synuclein (in-house produced αS)

Recombinant αS plasmid was obtained as a gift from Dr. Lapidus (Michigan State University). The PUC19/18 αS plasmid that carries human αS complementary DNA (cDNA) was transformed into competent cells (Rosetta BL21) on ampicillin-coated agar plates. A colony was used for a small bacteria culture (10 mL LB medium supplemented with 50 µg/mL ampicillin) and then left to shake overnight at 37 °C. The small culture was transferred to a 1 L culture of *E*. *coli* and grown for 3 h with shaking. The expression of the protein was induced with the addition of 1 mM isopropylthio-β-galactosidase (IPTG) after the larger culture reached an OD_600_ 0.5-0.7. After 6 h, cells were centrifuged to obtain the pellet at 6,600 g for 15 min at 4 ºC and then resuspended in lysis buffer (10 mM Tris pH 7.6, 750 mM NaCl, 1 mM EDTA with protease inhibitor). The lysates were subjected to sonication for 5 min (30 s on, 30 s off pulses at 30-45% power) and boiled at 95 °C for 15 min. The boiled sample was then centrifuged at 6,600 g for 15 min at 4 ºC and the supernatant obtained was transferred to a new tube, followed by overnight dialysis with 10 mM Tris pH 7.6, 1 mM ethylenediaminetetraacetic acid (EDTA). The dialyzed supernatant was purified by anion exchange chromatography (HiTrap Q HP 16/10) using a mobile phase of 25 mM Tris pH 7.6 and a linear gradient of 20 column volumes of elution buffer to 1 M NaCl. Fractions with αS were pooled and concentrated. The concentrated fraction was stored at -80 °C.

## Proteins

Recombinant human αS (commercial) (MW 14460 Da) and recombinant human αS with the A53T mutation (mαS, MW 14490 Da) were purchased from rPeptide (Watkinsville, GA, USA). Protein stock solutions, including recombinant human αS in-house produced, were diafiltrated against ultrapure water four times (4x) for 15 minutes, and then one more time for 45 minutes at 4 °C/14000 g using an Amicon 0.5 mL microconcentrator with a MW cutoff of 3 kDa (EMD Millipore, Billerica, MA). The desalted stocks were stored at -80 °C for long-term storage (weeks) and at −20 °C for short-term storage. The amount of protein was measured using the bicinchoninic acid (BCA) assay. A spectrophotometer Varioskan LUX (ThermoScientific, Singapore) at 562 nm (emission wavelength) was employed for fluorescence detection.

## Mass Spectrometry

Experiments were performed on a SCIEX 5600 hybrid quadrupole/time-of-flight tandem mass spectrometer (SCIEX, ON, Canada) modified for dipolar direct current (DDC) applications.^1^ Two gas phase collisional heating methods, executed sequentially, aid in mitigating metal ion adduction to protein ions. First, beam-type collision-induced dissociation (BTCID) is carried out by accelerating the ions into a quadrupole collision cell containing N_2_ bath gas (6-10 mTorr). Second, ions are displaced from the center of the ion trap by applying a DDC potential across opposing rods of the linear quadrupole ion trap. This translates the ions into regions of higher quadrupolar field strength, which increases their velocities due to radiofrequency-heating and thereby increases their collision energies with the bath gas.^2,3^ The latter event is a broadband activation method, i.e., all ions in the linear ion trap subjected to DDC are displaced to regions of higher quadrupolar field strength, in contrast with traditional single-frequency resonance excitation,^4^ which is a narrow-band activation method.

To perform nano-ESI (nESI), dual platinum wires supported by a single holder were inserted into the open ends of the theta emitter, with each wire contacting the solution inside the respective channels (two channels per theta emitter). Voltages of 0.80 – 1.8 kV were applied to the platinum wires to generate an electrospray, starting at 800 V and progressively increasing the voltage (waiting up to 7 scans) in intervals of 50 V (100 V increments after 1.0 kV) until the analyte ions were observed. Theta emitters were produced from borosilicate glass capillaries of 1.5 mm outer diameter (o.d.), 1.17 mm i.d., 10 cm length (Sutter Instruments, CA, USA, Part Number BT-150-10). The borosilicate glass capillaries were mechanically pulled with a P-87 micropipette puller (Sutter Instruments, CA, USA). The emitters were placed at ~1-2 mm away from the curtain plate, orthogonal to the MS orifice with a zero-degree angle with respect to the axis coming out of the orifice. Solutions of αS dissolved in biological buffers were loaded in one side of the theta emitters, while the other side was loaded with 199 mM ammonium acetate (AmAc) containing 1 mM ammonium bromide (AmBr) as a solution additive.^5^ BTCID was effected by accelerating ions into the collision cell (q2) and DDC was performed after ions were accumulated in q2, followed by time-of-flight mass analysis. Data acquisition and processing were performed identically for each experiment using custom software (SCIEX, ON, Canada) and PeakView (version 1.2.2.0), respectively. The spectra shown represent integration of several scans, only the duration of the electrospray yielding resolved charge states was considered for integration. No normalization was performed, but a smoothing step was included to convolute the ion signal of the free protein with the ion signal of the protein adducted to metal ions. Excel (version 2503) was used to create the plots of relative abundances (**Equation S1**), the ion intensities of the centroids at each charge state in the spectra from individual replicates was considered. All spectra were collected in n = 3 independent replicates. All ion peaks had a signal-to-noise ratio ≥ 5, each spectrum was manually inspected.

## Thioflavin T (ThT) fluorescence assay

A 96-well microplate was utilized for the experiment and the experimental procedure has been previously described.^6,7^ αS was introduced to each well to achieve a final concentration of 2 μM, from a stock solution of 277 μM, prepared by dissolving it in 20 mM Tris–HCl (pH 7.4). The assay conditions also included 10 mM PBS buffer (pH 7.4), 300 mM NaCl, 0.5 mM SDS, and a 3 mm borosilicate bead. ThT was added at a final concentration of 40 μM. The 96-well plate was loaded into a Synergy HT multi-mode microplate reader (BioTek, Winooski, VT) and incubated at 37 °C. The ThT fluorescence intensity was monitored with excitation at 440 nm and emission at 485 nm. Measurements were taken every 20 minutes with the plate shaken for 10 seconds before each reading. The kinetics were observed over a period of 42 to 50 h with each sample analyzed in triplicate.

## Equation

Equation S1

The relative abundance of the ion peaks in the spectrum was calculated using the following equation:

$$Relative abundance= \frac{I_{n}}{\sum_{1}^{n} I_{\alpha S}}$$

where *I_n_* represents the intensity of the *n* charge state (ion peaks with signal-to-noise ≥ 5), divided by the sum of the intensities of each of the charge states considered for the calculation ($\sum_{1}^{n} I_{\alpha S})$. Each spectrum was manually inspected, therefore, the number of charge states considered for each individual replicate might differ.

## Scheme


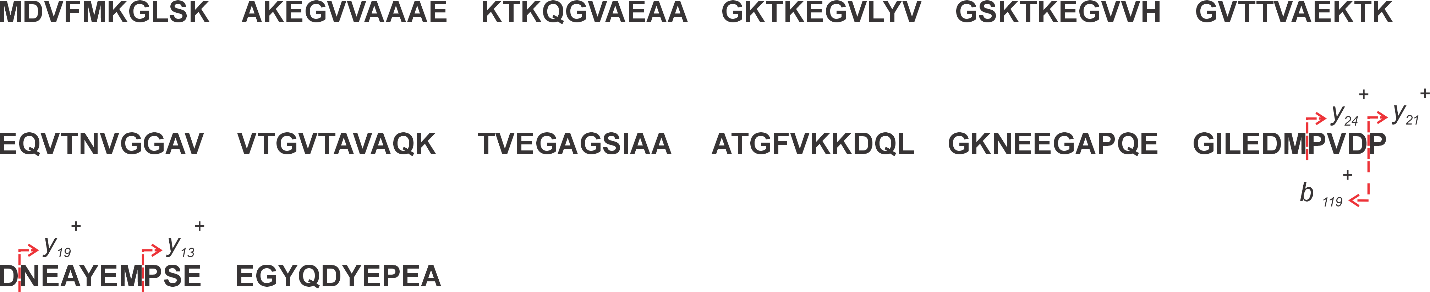


Scheme S1. Amino acid sequence of A53T α-synuclein (mαS). The *y* ions annotated in the spectra are shown in the sequence using red arrows. The complementary ion *b_119_*^+^ is also shown.

# Figures


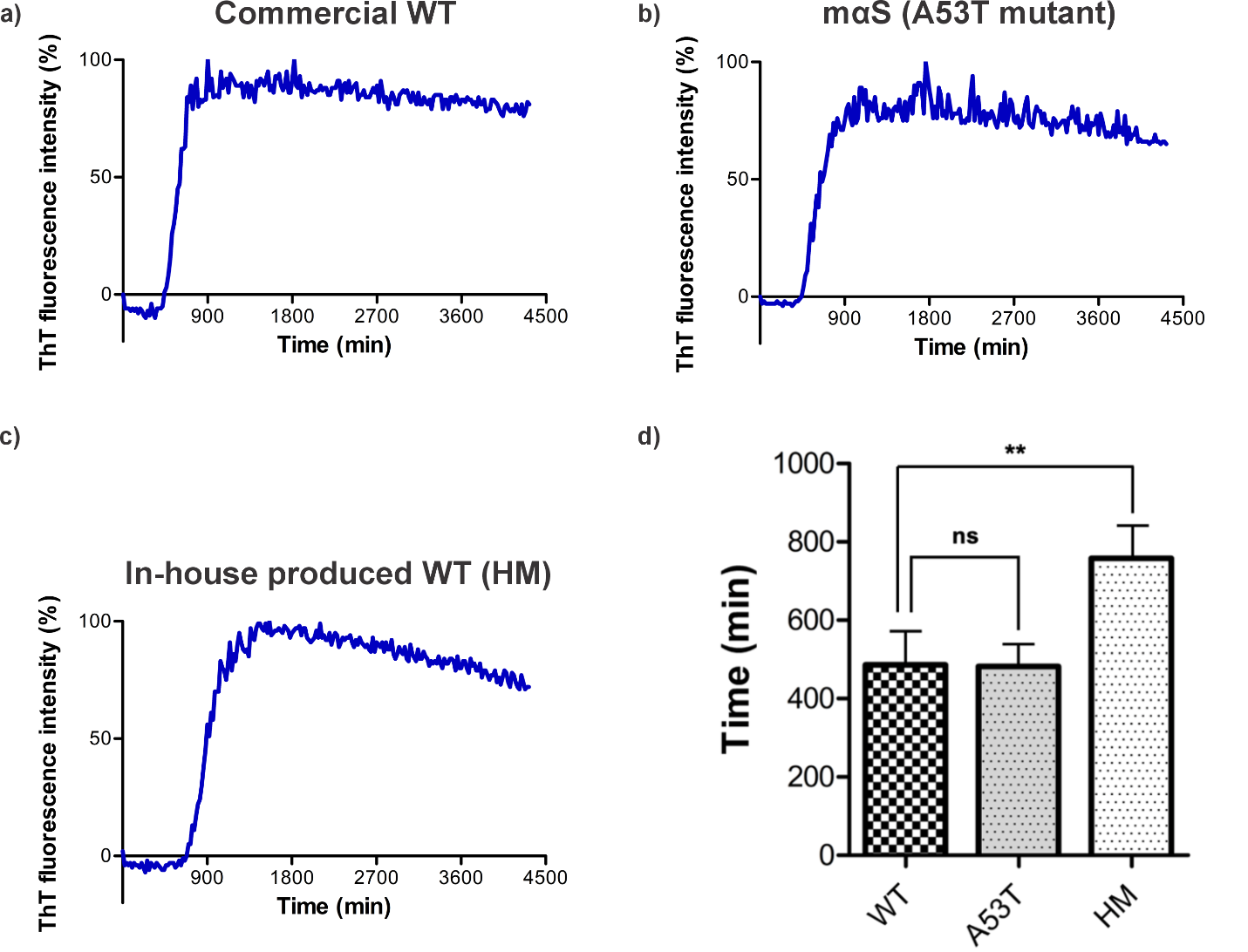


Figure S1**.** **Mutant (A53T) αS is more prone to aggregation in comparison with WT αS.** a) Kinetics of fibril formation of 2 μM a) WT αS with a lag time of 486.2 ± 38.5 minutes, b) A53T mutant αS (mαS) with a lag time of 481.9 ± 13.2 minutes, c) in-house produced (home-made, HM) WT αS with a lag time of 757.7 ± 24.5 minutes, d) *t*_lag_ bars plot. *t*_lag_ is the time before observing any ThT intensity increase. A Tukey’s multiple comparison test was performed to assess statistical significance differences between the samples. ns = not significant, ** means p < 0.001 (statistically significant). The Thioflavin T (ThT) fibrillation kinetics were acquired in the presence of 0.25% DMSO in a buffer comprised of 10 mM (1X) PBS pH 7.4 supplemented with 300 mM NaCl and 0.5 mM SDS. Each curve represents the average data from three replicates conducted within the same experiment.


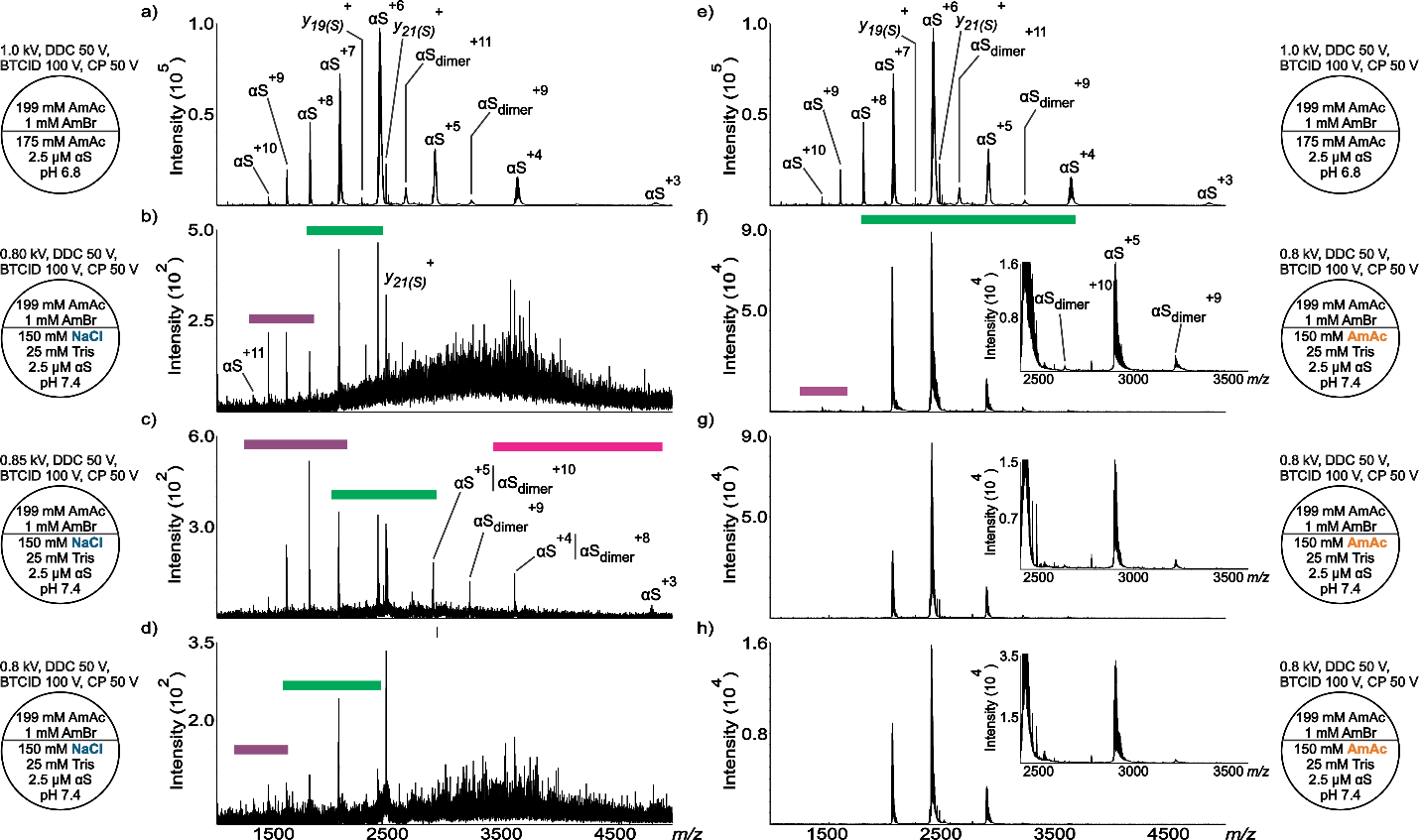


Figure S2. nESI mass spectra acquired in positive ion mode for aqueous solutions of commercial a) αS (2.5 μM) dissolved in 175 mM AmAc pH 6.8, b-d) αS (2.5 μM) dissolved in 150 mM NaCl plus 25 mM Tris pH 7.4, e) same spectrum as “a”, f-h) αS (2.5 μM) dissolved in 150 mM AmAc plus 25 mM Tris pH 7.4. The circle split by half represents a theta emitter. The theta emitters were all loaded with 199 mM AmAc plus 1 mM AmBr in the opposite side. All solutions were prepared individually for n = 3 independent replicates.


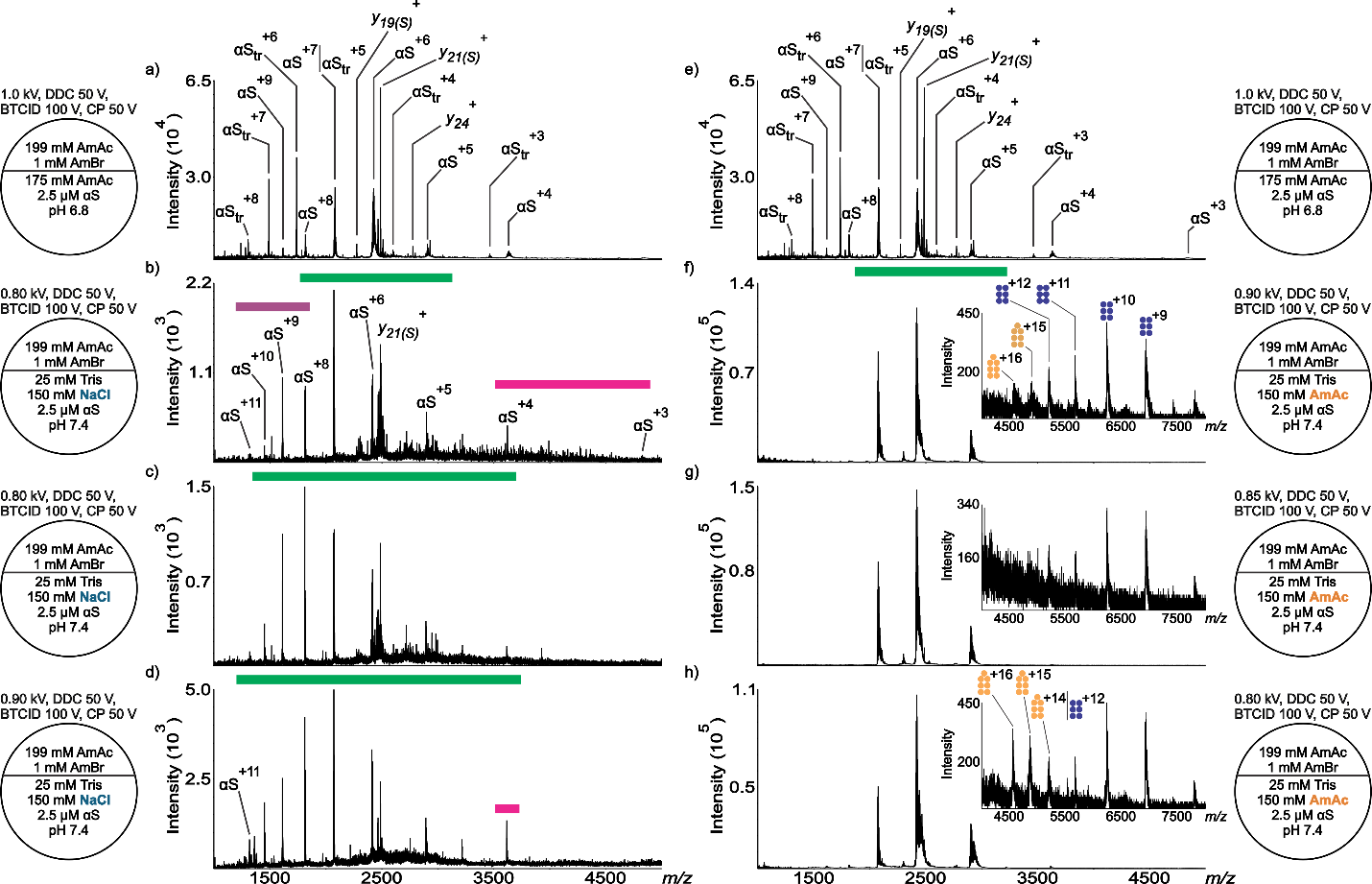


Figure S3. nESI mass spectra acquired in positive ion mode for aqueous solutions of in-house produced a) αS (2.5 μM) dissolved in 175 mM AmAc pH 6.8, b-d) αS (2.5 µM) dissolved in 150 mM NaCl plus 25 mM Tris pH 7.4, e) same spectrum as “a”, f-h) αS (2.5 μM) dissolved in 150 mM AmAc plus 25 mM Tris pH 7.4. The circle split by half represents a theta emitter. The theta emitters were all loaded with 199 mM AmAc plus 1 mM AmBr in the opposite side. All solutions were prepared individually for n = 3 independent replicates. Hexamer of truncated αS (αS_tr_) is represented in blue, while the heptamer of αS_tr_ is represented in light orange.


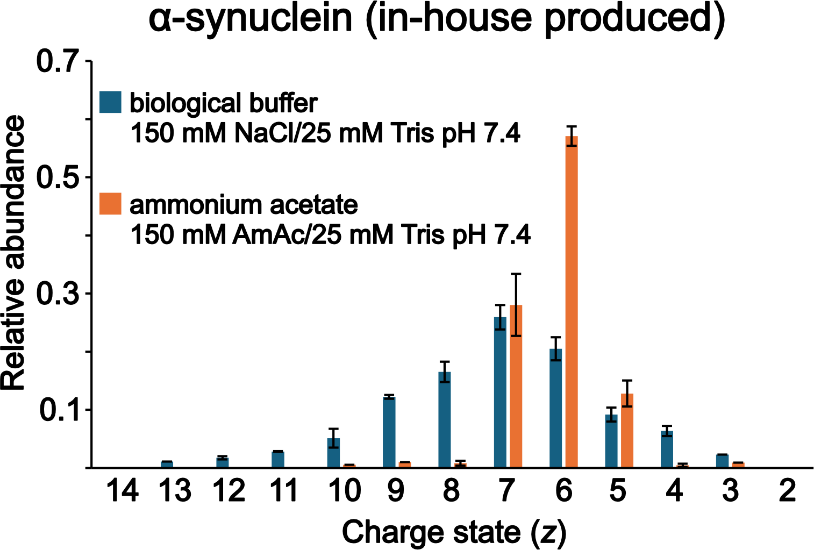


Figure S4. Relative abundances of the mαS_monomer_ ion peaks shown in Figure S3.


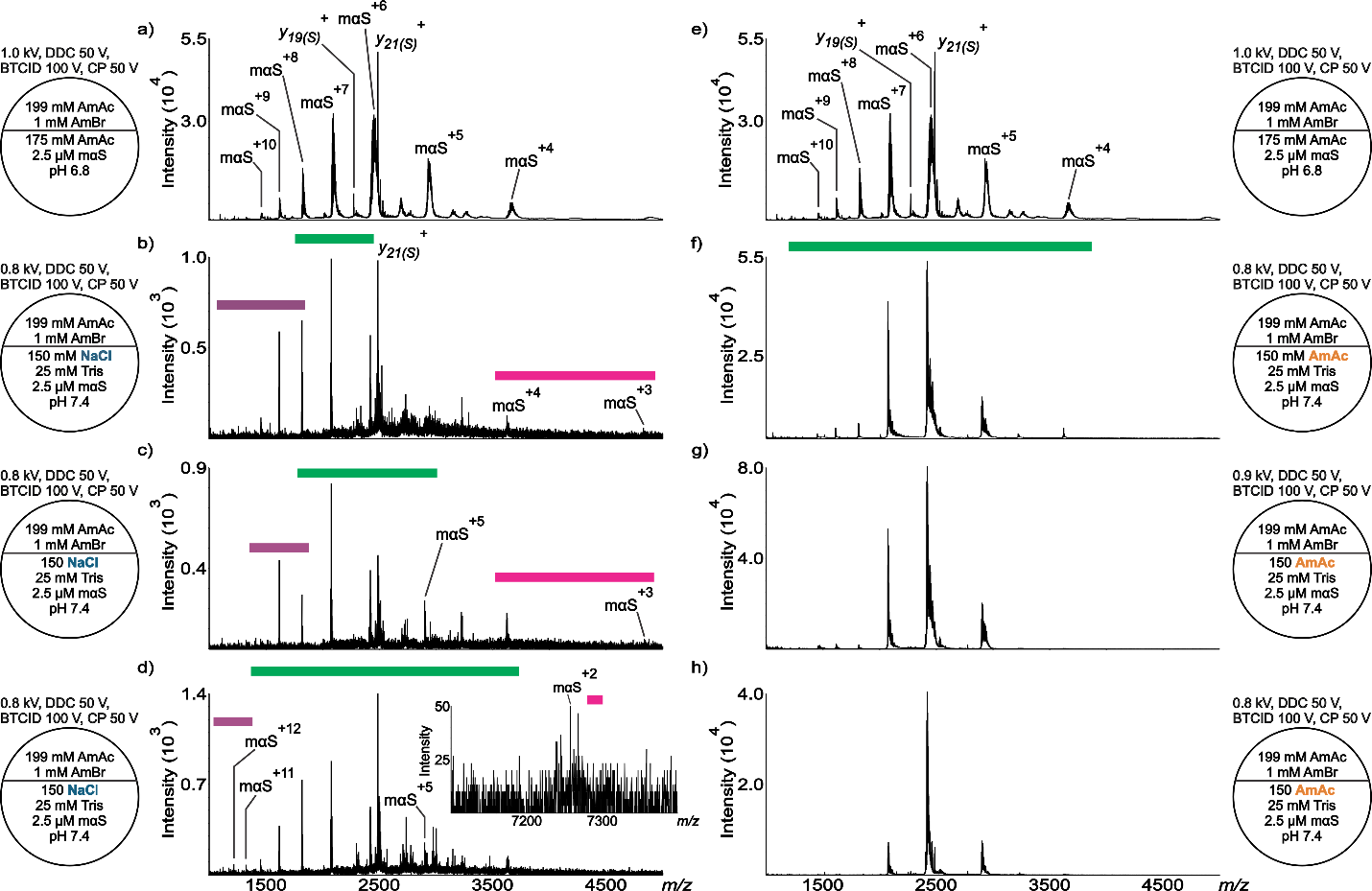


Figure S5. nESI mass spectra acquired in positive ion mode for aqueous solutions of a) mαS (2.5 μM) dissolved in 175 mM AmAc pH 6.8, b-d) mαS (2.5 μM) dissolved in 150 mM NaCl plus 25 mM Tris pH 7.4, e) same spectrum as “a”, f-h) mαS (2.5 μM) dissolved in 150 mM AmAc plus 25 mM Tris pH 7.4. The circle split by half represents a theta emitter. The theta emitters were all loaded with 199 mM AmAc plus 1 mM AmBr in the opposite side. All solutions were prepared individually for n = 3 independent replicates. There are other poorly resolved ion peaks in the spectra of mαS dissolved in 25 mM Tris/150 mM NaCl, which could not be assigned.


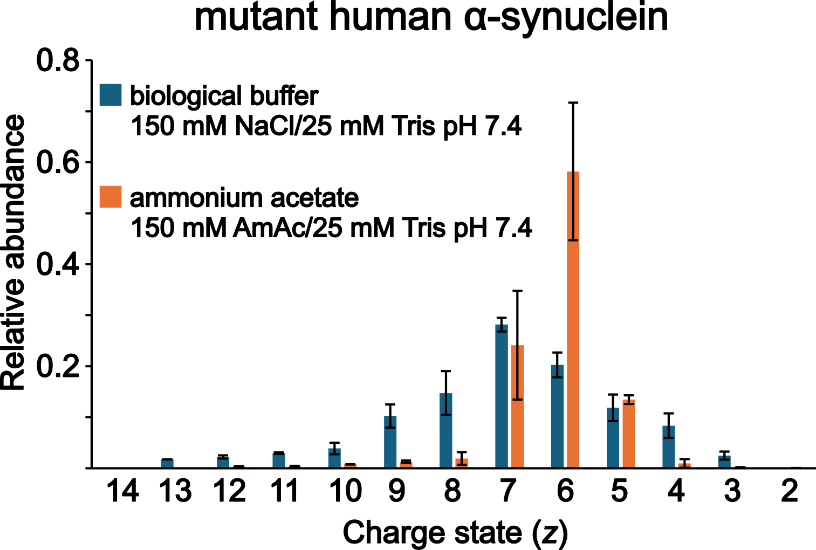


Figure S6. Relative abundances of the mαS_monomer_ ion peaks shown in Figure S5.


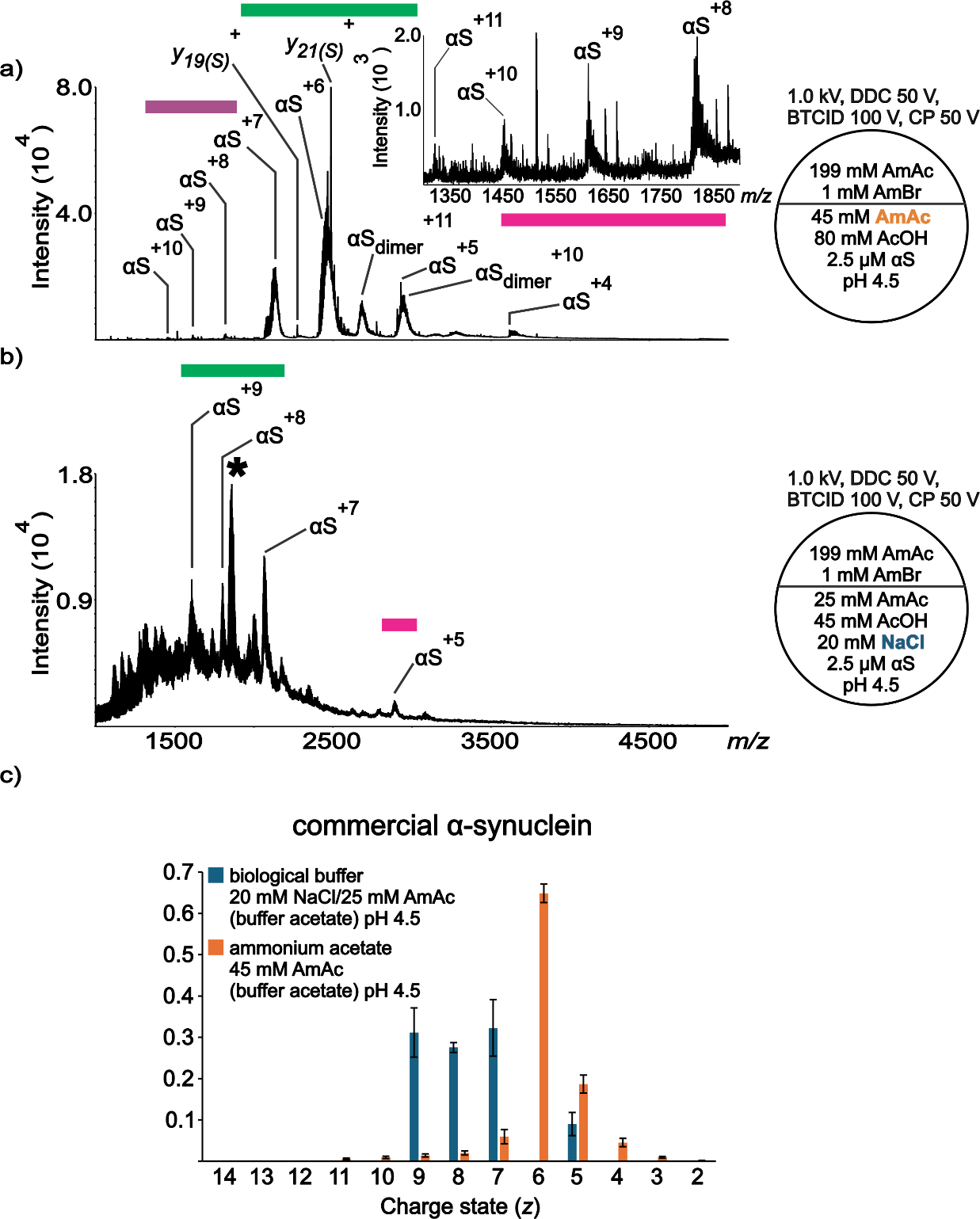


Figure S7. nESI mass spectra acquired in positive ion mode for aqueous solutions of commercially available a) αS (2.5 μM) dissolved in 45 mM AmAc plus 80 mM AcOH pH 4.5, b) αS (2.5 μM) dissolved in 20 mM NaCl, 25 mM AmAc plus 45 mM AcOH pH 4.5, c) relative abundances of the ion peaks observed in “a” and “b”. Three independent replicates were taken into consideration. The circle split by half represents a theta emitter. The band highlighted in blue points out the absence of αS^+6^. The asterisk signals unresolved features in the spectra, therefore, no charge state was assigned due to high uncertainty.


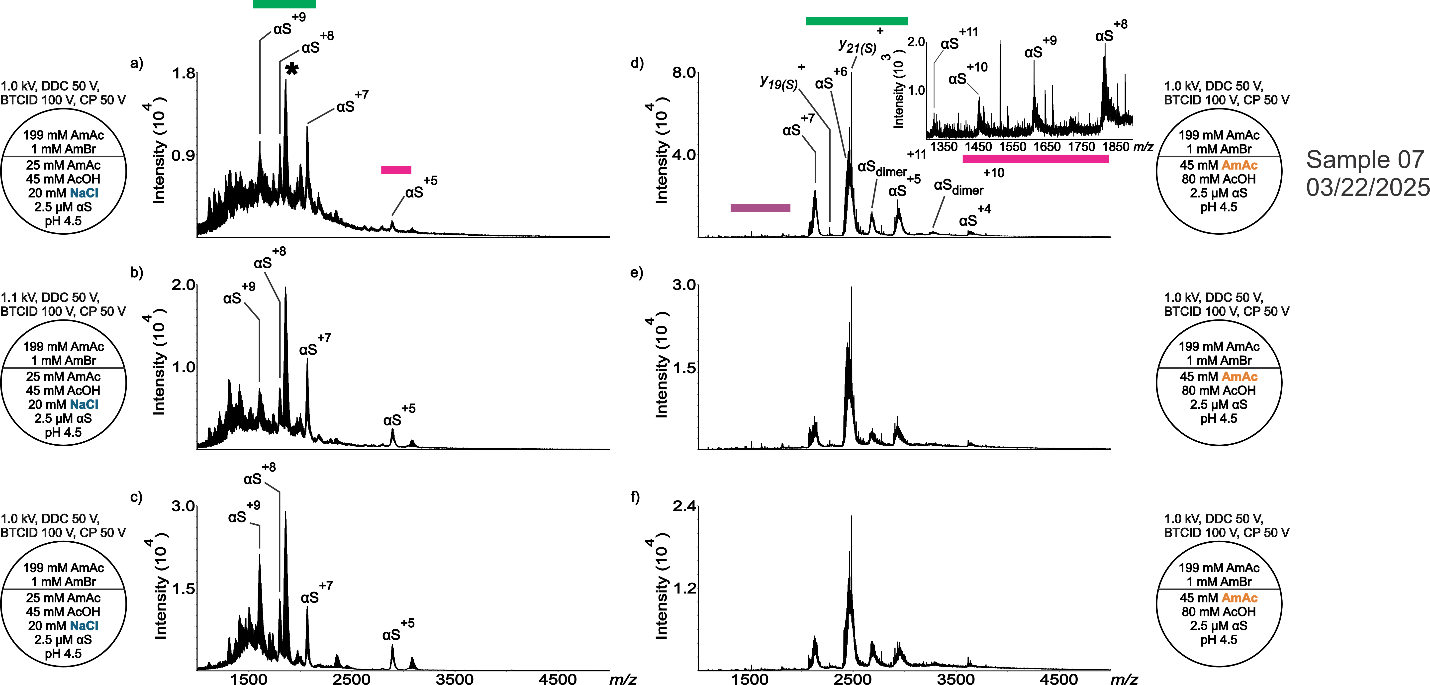


Figure S8. nESI mass spectra acquired in positive ion mode for aqueous solutions of commercially available a-c) αS (2.5 μM) dissolved in 25 mM AmAc, 45 mM AcOH plus 20 mM NaCl pH 4.5, d-f) αS (2.5 μM) dissolved in 45 mM AmAc plus 80 mM AcOH pH 4.5. The circle split by half represents a theta emitter. The theta emitters were all loaded with 199 mM AmAc plus 1 mM AmBr in the opposite side. All solutions were prepared individually for n = 3 independent replicates. The asterisk signals unresolved features in the spectra, therefore, no charge state was assigned due to high uncertainty.


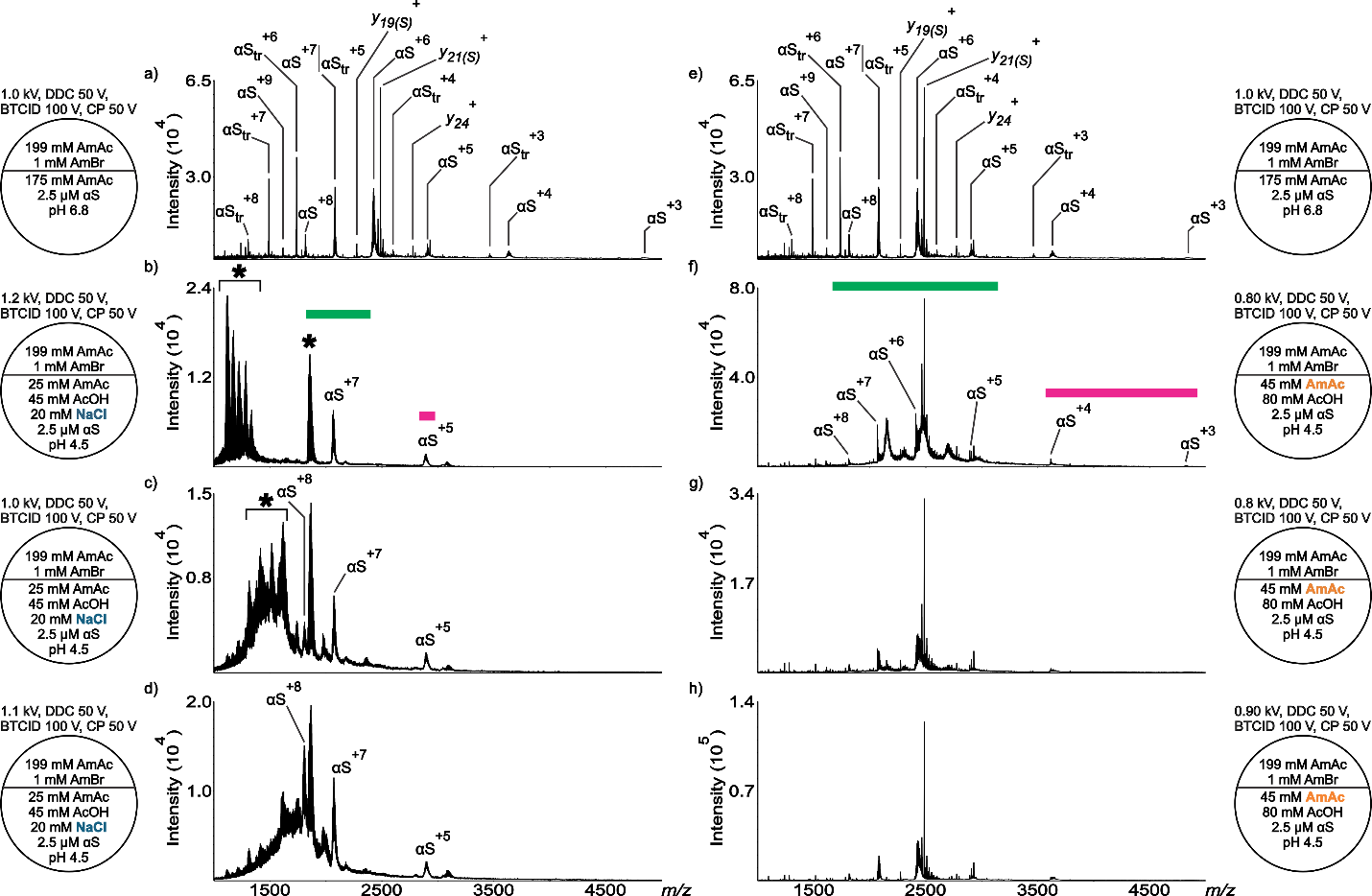


Figure S9. nESI mass spectra acquired in positive ion mode for aqueous solutions of in-house produced a) αS (2.5 μM) dissolved in 175 mM AmAc pH 6.8, b-d) αS (2.5 µM) dissolved in 25 mM AmAc, 45 mM AcOH plus 20 mM NaCl pH 4.5, e) same spectrum as “a”, f-h) αS (2.5 μM) dissolved in 45 mM AmAc plus 80 mM AcOH pH 4.5. The circle split by half represents a theta emitter. The theta emitters were all loaded with 199 mM AmAc plus 1 mM AmBr in the opposite side. All solutions were prepared individually for n = 3 independent replicates. The asterisk signals unresolved features in the spectra, therefore, no charge state was assigned due to high uncertainty.


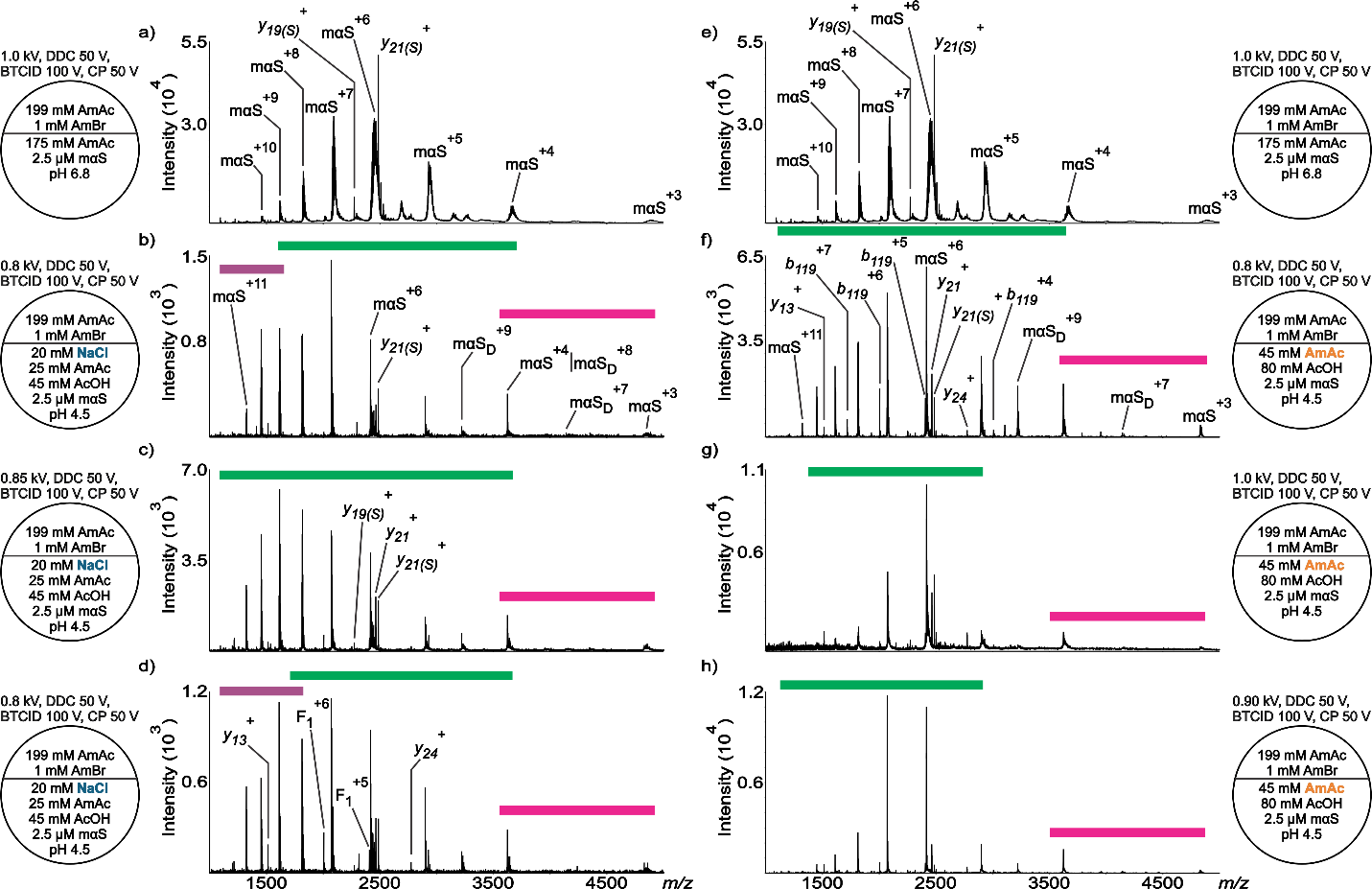


Figure S10. nESI mass spectra acquired in positive ion mode for aqueous solutions of a) mαS (2.5 μM) dissolved in 175 mM AmAc pH 6.8, b-d) mαS (2.5 µM) dissolved in 25 mM AmAc, 45 mM AcOH plus 20 mM NaCl pH 4.5, e) same spectrum as “a”, f-h) mαS (2.5 μM) dissolved in 45 mM AmAc plus 80 mM AcOH pH 4.5. The circle split by half represents a theta emitter. The theta emitters were all loaded with 199 mM AmAc plus 1 mM AmBr in the opposite side. All solutions were prepared individually for n = 3 independent replicates.


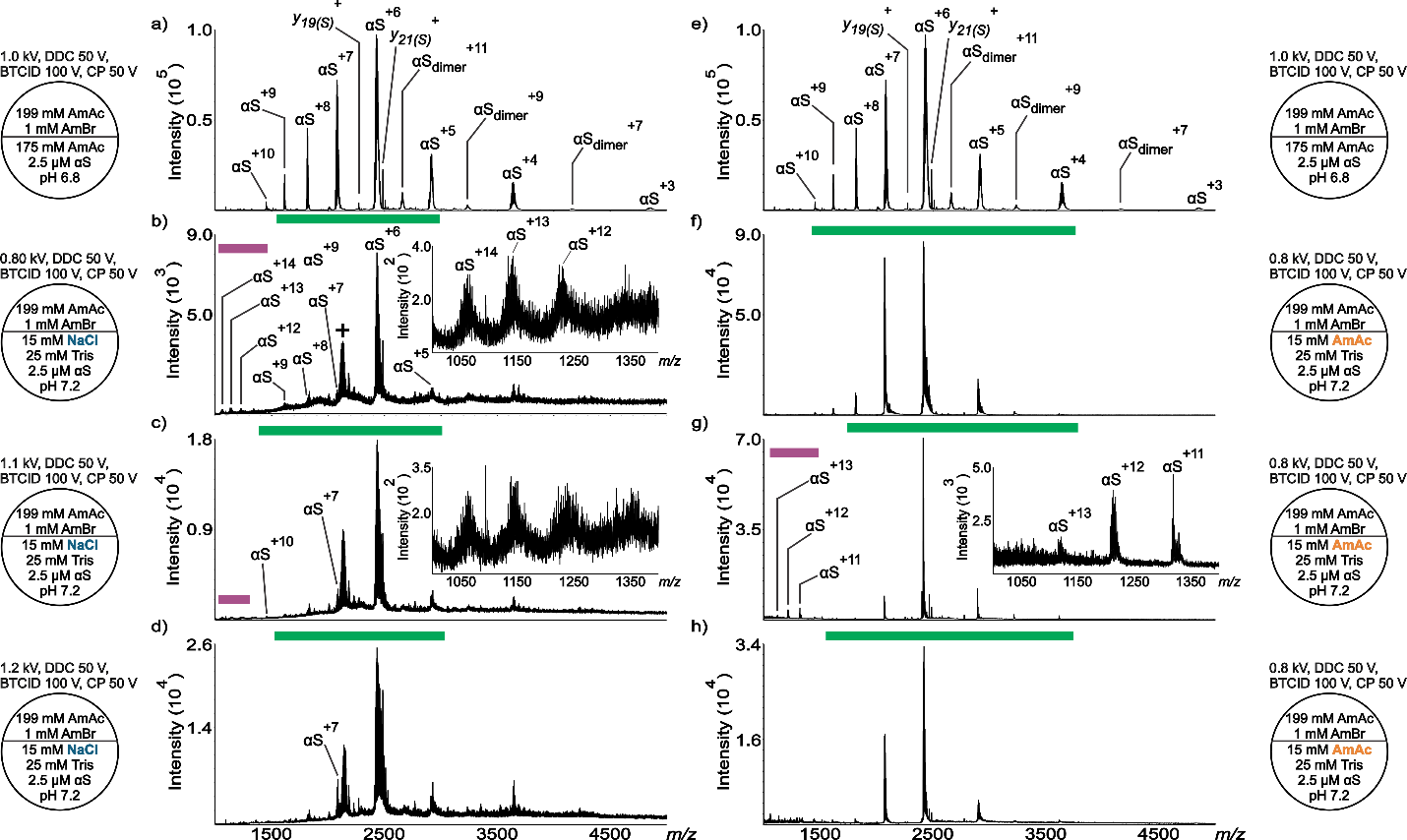


Figure S11. nESI mass spectra acquired in positive ion mode for aqueous solutions of commercially available a) αS (2.5 µM) dissolved in 175 mM AmAc pH 6.8, b-d) αS (2.5 μM) dissolved in 15 mM NaCl plus 25 mM Tris pH 7.2, e) same spectrum as “a”, f-h) αS (2.5 μM) dissolved in 15 mM AmAc plus 25 mM Tris pH 7.2. The circle split by half represents a theta emitter. The theta emitters were all loaded with 199 mM AmAc plus 1 mM AmBr in the opposite side. All solutions were prepared individually for n = 3 independent replicates. The symbol (+) represents an unresolved relatively high intensity ion peak.


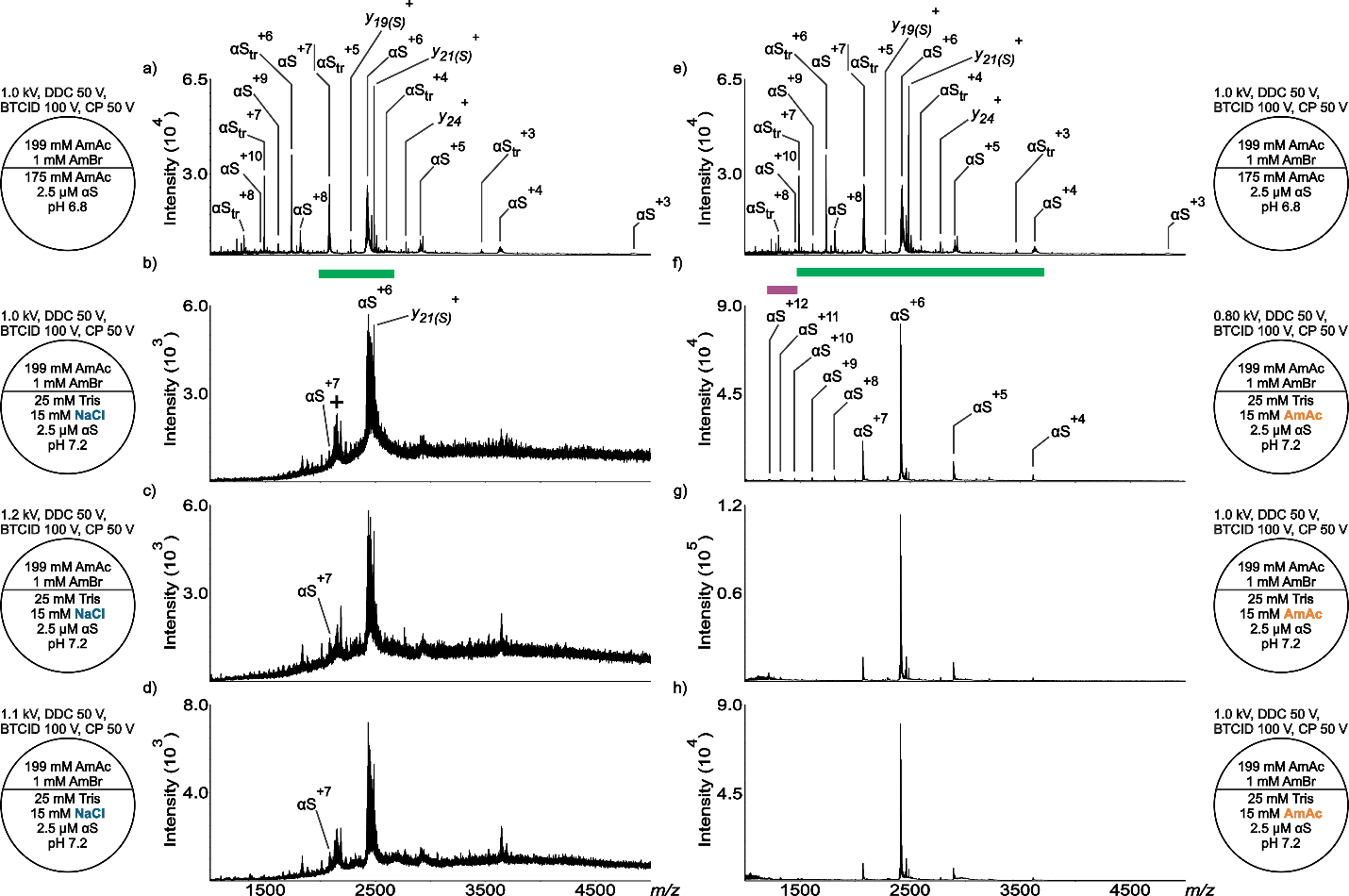


Figure S12. nESI mass spectra acquired in positive ion mode for aqueous solutions of in-house produced a) αS (2.5 μM) dissolved in 175 mM AmAc pH 6.8, b-d) αS (2.5 µM) dissolved in 15 mM NaCl plus 25 mM Tris pH 7.2, e) same spectrum as “a”, f-h) αS (2.5 μM) dissolved in 15 mM AmAc plus 25 mM Tris pH 7.2. The circle split by half represents a theta emitter. The theta emitters were all loaded with 199 mM AmAc plus 1 mM AmBr in the opposite side. All solutions were prepared individually for n = 3 independent replicates. The symbol (+) represents an unresolved relatively high intensity ion peak.


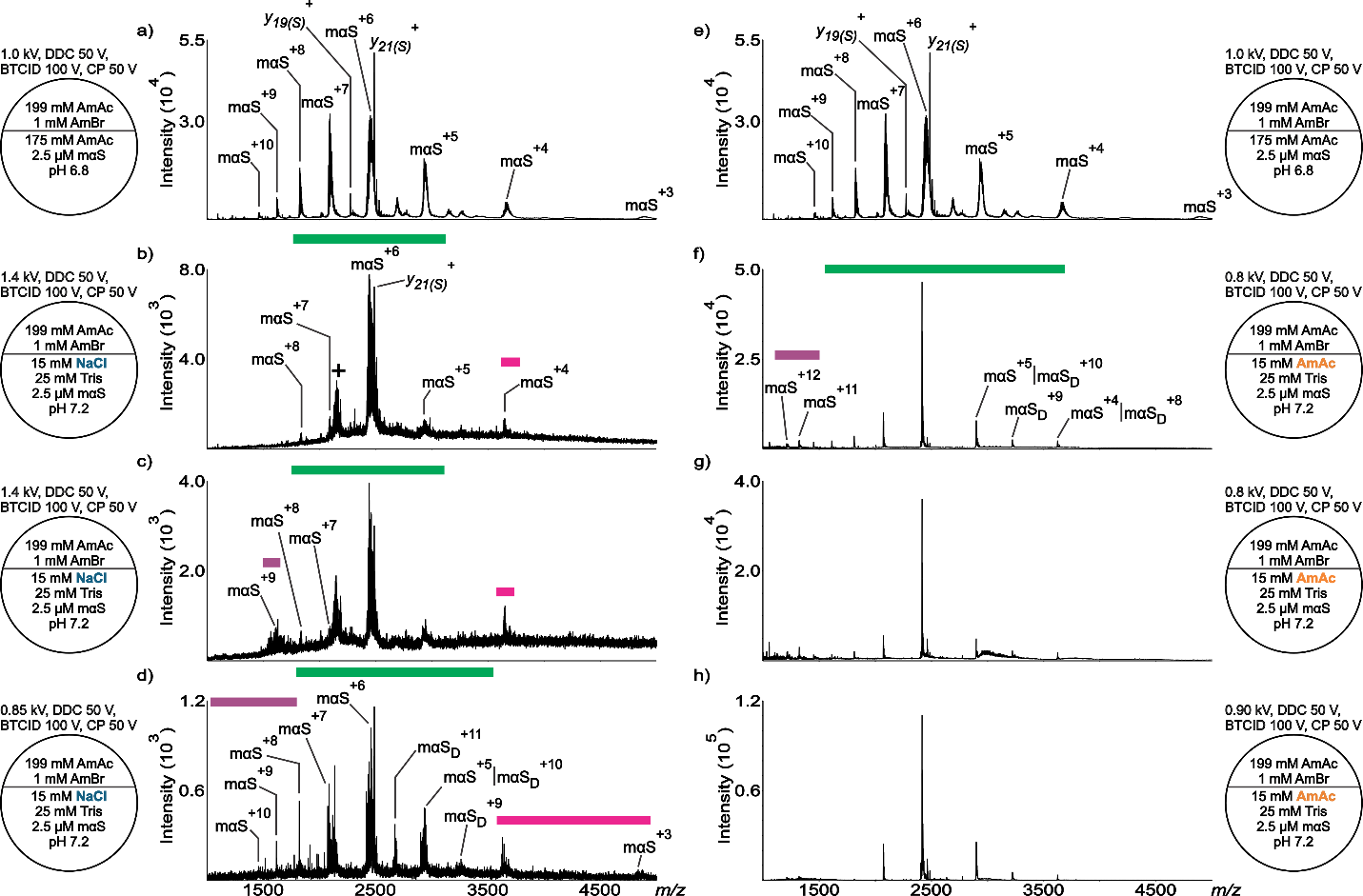


Figure S13. nESI mass spectra acquired in positive ion mode for aqueous solutions of a) mαS (2.5 μM) dissolved in 175 mM AmAc pH 6.8, b-d) mαS (2.5 µM) dissolved in 15 mM NaCl plus 25 mM Tris pH 7.2, e) same spectrum as “a”, f-h) mαS (2.5 μM) dissolved in 15 mM AmAc plus 25 mM Tris pH 7.2. The circle split by half represents a theta emitter. The theta emitters were all loaded with 199 mM AmAc plus 1 mM AmBr in the opposite side. All solutions were prepared individually for n = 3 independent replicates. The symbol (+) represents an unresolved relatively high-intensity ion peak.

## References

(1) Bhanot, J. S.; Fabijanczuk, K. C.; Abdillahi, A. M.; Chao, H. C.; Pizzala, N. J.; Londry, F. A.; Dziekonski, E. T.; Hager, J. W.; McLuckey, S. A. Adaptation and Operation of a Quadrupole/Time-of-Flight Tandem Mass Spectrometer for High Mass Ion/Ion Reaction Studies. *Int. J. Mass Spectrom.* **2022**, *478*.

(2) Tolmachev, A. V.; Vilkov, A. N.; Bogdanov, B.; PĂsa-Tolić, L.; Masselon, C. D.; Smith, R. D. Collisional Activation of Ions in RF Ion Traps and Ion Guides: The Effective Ion Temperature Treatment. *J. Am. Soc. Mass Spectrom.* **2004**, *15* (11), 1616–1628.

(3) Mehnert, S. A.; Fischer, J. L.; McDaniel, M. R.; Fabijanczuk, K. C.; McLuckey, S. A. Dissociation Kinetics in Quadrupole Ion Traps: Effective Temperatures under Dipolar DC Collisional Activation Conditions. *J. Am. Soc. Mass. Spectrom.* **2023**, *34* (6), 1166–1174.

(4) Louris, J. N.; Cooks, R. Graham.; Syka, J. E. P.; Kelley, P. E.; Stafford, G. C.; Todd, J. F. J. Instrumentation, Applications, and Energy Deposition in Quadrupole Ion-Trap Tandem Mass Spectrometry. *Anal. Chem.* **1987**, *59* (13), 1677–1685.

(5) Báez Bolívar, E. G.; McLuckey, S. A. Improved Mass Analysis of Proteins and Protein Complexes Present in Common Biological Buffers and Physiological Concentrations of NaCl Using Solution Additives Delivered by Theta Emitters. *Anal. Chem.* **2025**, *97* (38), 20859–20868.

(6) Fortin, J. S.; Hetak, A. A.; Duggan, K. E.; Burglass, C. M.; Penticoff, H. B.; Schott, H. C. Equine Pituitary Pars Intermedia Dysfunction: A Spontaneous Model of Synucleinopathy. *Sci. Rep.* **2021**, *11* (1), 16036.

(7) Ramirez, E.; Ganegamage, S. K.; Elbatrawy, A. A.; Alnakhala, H.; Shimanaka, K.; Tripathi, A.; Min, S.; Rochet, J. C.; Dettmer, U.; Fortin, J. S. 5-Nitro-1,2-Benzothiazol-3-Amine and N-Ethyl-1-[(Ethylcarbamoyl)(5-Nitro-1,2-Benzothiazol-3-Yl)Amino]Formamide Modulate α-Synuclein and Tau Aggregation. *ACS Omega* **2023**, *8* (22), 20102–20115.
